# Facile adipocyte uptake and liver/adipose tissue delivery of conjugated linoleic acid-loaded tocol nanocarriers for a synergistic anti-adipogenesis effect

**DOI:** 10.1186/s12951-024-02316-8

**Published:** 2024-02-06

**Authors:** Ching-Yun Hsu, Chia-Chih Liao, Zih-Chan Lin, Ahmed Alalaiwe, Erica Hwang, Tzu-Wei Lin, Jia-You Fang

**Affiliations:** 1 Department of Nutrition and Health Sciences, Chang Gung University of Science and Technology, Kweishan, Taoyuan, Taiwan; 2 Research Center for Food and Cosmetic Safety and Research Center for Chinese Herbal Medicine, Chang Gung University of Science and Technology, Kweishan, Taoyuan, Taiwan; 3 Department of Anesthesiology, Chang Gung Memorial Hospital at Linkou, Taoyuan, Taiwan; 4 School of Medicine, College of Medicine, Chang Gung University, Taoyuan, Taiwan; 5 Chronic Diseases and Health Promotion Research Center, Chang Gung University of Science and Technology, Puzi, Chiayi, Taiwan; 6 Department of Pharmaceutics, College of Pharmacy, Prince Sattam Bin Abdulaziz University, Al Kharj, Saudi Arabia; 7 Department of Dermatology, Yale School of Medicine, Yale University, New Haven, CT USA; 8 Pharmaceutics Laboratory, Graduate Institute of Natural Products, Chang Gung University, 259 Wen-Hwa 1st Road, Kweishan, Taoyuan, 333 Taiwan

**Keywords:** Conjugated linoleic acid, α-tocopherol, Nanostructured lipid carriers, Passive targeting, Anti-adipogenesis, Obesity

## Abstract

Obesity is a major risk to human health. Adipogenesis is blocked by α-tocopherol and conjugated linoleic acid (CLA). However, their effect at preventing obesity is uncertain. The effectiveness of the bioactive agents is associated with their delivery method. Herein, we designed CLA-loaded tocol nanostructured lipid carriers (NLCs) for enhancing the anti-adipogenic activity of α-tocopherol and CLA. Adipogenesis inhibition by the nanocarriers was examined using an in vitro adipocyte model and an in vivo rat model fed a high fat diet (HFD). The targeting of the tocol NLCs into adipocytes and adipose tissues were also investigated. A synergistic anti-adipogenesis effect was observed for the combination of free α-tocopherol and CLA. Nanoparticles with different amounts of solid lipid were developed with an average size of 121‒151 nm. The NLCs with the smallest size (121 nm) showed greater adipocyte internalization and differentiation prevention than the larger size. The small-sized NLCs promoted CLA delivery into adipocytes by 5.5-fold as compared to free control. The nanocarriers reduced fat accumulation in adipocytes by counteracting the expression of the adipogenic transcription factors peroxisome proliferator activated receptor (PPAR)γ and CCAAT/enhancer-binding protein (C/EBP)α, and lipogenic enzymes acetyl-CoA carboxylase (ACC) and fatty acid synthase (FAS). Localized administration of CLA-loaded tocol NLCs significantly reduced body weight, total cholesterol, and liver damage indicators in obese rats. The biodistribution study demonstrated that the nanoparticles mainly accumulated in liver and adipose tissues. The NLCs decreased adipocyte hypertrophy and cytokine overexpression in the groin and epididymis to a greater degree than the combination of free α-tocopherol and CLA. In conclusion, the lipid-based nanocarriers were verified to inhibit adipogenesis in an efficient and safe way.

**Supplementary Information:**

The online version contains supplementary material available at 10.1186/s12951-024-02316-8.

## Introduction

Obesity is associated with cardiovascular disease, hypertension, diabetes, and cancers, accounting for > 70% of early deaths [1]. About one-third of the global population is classified as obesity or overweight [2]. Obesity also shortens the life span by 5‒20 years, and causes the dysfunction of many organs [3]. Adipocytes have a vital role on obesity development. Adipocytes are endocrine cells that maintain energy balance by triglyceride (TG) storage and fatty acid release in response to nutritional signaling [4]. Obesity is associated with an increased size and number of adipocytes. Adipogenesis inhibition and lipolysis induction in adipocytes are the focus of methods for treating obesity [5].

Current management of obesity includes dietary control, pharmacotherapy, intragastric balloon placement, and bariatric surgery [6]. However, these treatments can be associated with high cost, low effectiveness, and risks of complications. For instance, the long-term use of pharmacotherapy such as orlistat, phentermine, and lorcaserin is associated with headache, dizziness, and itching [7]. In addition, current pharmacotherapy insufficiently targets adipocytes and fat tissue, resulting in poor effectiveness. As such, new innovative methods to treat obesity in an efficient and safe way are needed. Nanocarriers may be a novel approach to overcome the disadvantages of the current anti-obesity drugs. Drugs can be loaded into nanoparticles to increase their solubility, stability, and bioavailability. Controlled drug release and local targeting of action can be achieved by nanoencapsulation, thus the drug can be specifically delivered into the target tissue, and the dose can be reduced to minimize side effects [8].

The development of the new agents from natural compounds is another focus of anti-obesity research. Conjugated linoleic acid (CLA) is reported to enhance weight loss and lean body mass [9]. CLA is a mixture of positional and geometric isomers of linoleic acid (LA) with conjugated double bonds. There are 28 isomers of CLA, mainly derived from meat and milk of ruminants [10]. The isomers *cis*-9, *trans*-11 (*c*9-*t*11) and *trans*-10, *cis*-12 (*t*10-*c*12) are the most abundant and have the greatest bioactivity [11]. Both compounds modulate fatty acid composition and adipokine metabolism to promote body fat reduction [12]. However, evidence of the anti-obesity activity of CLA is weaker in human studies than in animal studies [13]. This may be due to the need of a high dose of CLA, and poor adipocyte targeting [9].

Adipocyte-targeting is critical for the local reduction of adipose tissue. The entrapment of fatty acids in nano-formulations is an effective method for controlled delivery and for improving the drug bioactivity [14]. The incorporation of CLA with other anti-obesity agents in delivery systems is another approach to increase the drug effectiveness. There is evidence that α-tocopherol (vitamin E) has antioxidant and anti-cytokine activities, and can control energy balance and reduce body weight in animals and humans [15]. The primary source of α-tocopherol is diet, and is mainly found in natural oils, oil seeds, and nuts. As with CLA, α-tocopherol requires a high dose and long treatment duration to decrease body weight in humans. Although it is clear that adipocytes and adipose tissues are targets for anti-obesity treatments, there is no approved drug or formulation with selective targeting.

The purpose of this study was to design α-tocopherol-incorporated nanostructured lipid carriers (tocol NLCs) to encapsulate CLA for a synergistic targeting therapy for obesity. The conjugated double-bonds in CLA indicate a susceptibility for oxidative deterioration [16]. This effect can be suppressed by the antioxidant property of α-tocopherol, which is transported into and concentrated in adipose tissue [17]. In the design of tocol NLCs, α-tocopherol has 4 roles: (1) an adipogenesis inhibitor; (2) an oil to form lipid nanoparticles; (3) antioxidant protection of CLA; and (4) passive targeting to adipose tissue. NLCs are the second generation of solid lipid nanoparticles (SLNs), and the use of liquid oil together with the solid lipid in NLCs allows the formation of an amorphous matrix that improves drug loading capability, physical stability, and controlled release [18]. Lipid droplet formation and adipokine production are 2 characteristics of the adipogenesis of mature adipocytes, and 3T3-L1 pre-adipocytes are the most-used adipocyte-like phenotype to test the anti-adipogenesis effect of the bioactive compounds [19]. The hormonal cocktail activation can differentiate 3T3-L1 cells into adipocytes. In this study, 3T3-L1 cells were used to examine the in vitro bioactivity of the tocol NLCs, and the in vivo effects were examined using a HFD rat obesity model.

## Materials and methods

### Preparation of tocol NLCs

The lipid and aqueous phases of tocol NLCs were fabricated separately. The lipid phase of tocol NLCs with a small size consisted of 473 mg α-tocopherol, 31 mg CLA, 150 mg hexadecyl palmitate (HP), and 150 mg soybean phosphatidylcholine (SPC, Phospholipon 80 H). CLA was purchased from Sigma-Aldrich and was fabricated with an alkaline isomerization from LA-rich safflower oil, yielding 41.2% *c*9-*t*11 and *t*9-*c*11 CLA, 44.1% *t*10-*c*12 CLA, and 9.4% *t*10-*c*12 CLA. The HP amount was increased to 200 mg to produce NLCs with a medium particle size, and 297 mg to produce NLCs with a large particle size. The aqueous phase consisted of 400 mg Poloxamer 188 and water. Both phases were heated separately to 85 °C for 20 min. The aqueous phase was added into the lipid phase, then the mixture was homogenized with a high-shear homogenizer at 12,000 rpm for 20 min. The mixture was further homogenized using a probe-type sonicator at 35 W for 20 min. The final volume of the nanosystems was 10 ml. The molar ratio of α-tocopherol to CLA in the nanosystems was 10:1.

### Physicochemical characterization of tocol NLCs

The particle diameter, polydispersity index (PDI), and zeta potential were detected by dynamic light scattering (Nano ZS90, Malvern). The NLCs were diluted 100-fold with water before the measurement. The nanodispersion was centrifugated at 48,000 ×*g* at 4 °C for 30 min to remove the free CLA in the supernatant. The precipitate with nanoparticle-encapsulated CLA was dissolved by Triton X-100. The encapsulation efficiency of CLA was quantified by liquid chromatography/mass spectrometry (LC/MS), using a system that included a Dionex UltiMate 3000 equipped with a Thermo Finnigan LXQ linear ion trap mass spectrometer operated in positive electrospray ion mode. An Acquity UPLC C18 column was used, and maintained at 35 °C with a flow rate of 0.3 ml/min. The morphology of nanoparticles was seen by transmission electron microscopy (TEM). The nanodispersion (10 µl) was loaded onto a 3-mm Formvar/Carbon-supported copper grid. After a 3-min incubation, the excess water was soaked up. The sample was then placed in the oven for drying overnight. The samples were visualized with Hitachi HT7800 TEM. To evaluate the storage stability of tocol NLCs, the nanosystems were stored at room temperature in a desiccator for 21 days, and the relative humidity was maintained at about 50%. Aliquot of the samples were obtained at 7, 14, and 21 days to determine the particle size.

### 3T3-L1 cell differentiation

The 3T3-L1 pre-adipocytes were culture in DMEM containing 10% fetal bovine serum (FBS), 1% penicillin/streptomycin, and 1 mM sodium pyruvate in a 5% CO_2_ incubator at 37 °C. After 4 days, the medium was changed to differentiation-induction medium composed of DMEM containing 10% FBS, 500 µM 3-isobutyl-1-methylxanthine, 0.25 µM dexamethasone, and 10 µg/ml bovine insulin (MDI). After 2 days, the culture medium was replaced with DMEM containing 10% FBS and 10 µg/ml bovine insulin. The cells were incubated for the following 4 days to complete the differentiation process.

### Cell viability assay

The 3T3-L1 cells were seeded in 96-well microplates in DMEM at 1 × 10^5^ cells/well. α-Tocopherol, LA, or CLA (1‒500 µM) in free and nanoparticulate forms were added to the wells for a 24-h treatment. Then, 3-(4,5-dimethylthiazol-2-yl)-2,5-diphenyltetrazolium bromide (MTT) at 0.5 mg/ml was added to the wells for 2 h. The other procedures were the same as previously reported [20].

### Lipid droplet staining by oil red O (ORO)

Intracellular lipid deposition in 3T3-L1 cells was determined by ORO staining. The pre-adipocytes were seeded in 6-well microplates at 8 × 10^4^ cells/well. The cells were treated with α-tocopherol, LA, or CLA (10‒100 µM) in free or nanoparticulate forms at day 6 of differentiation. On day 8, the medium was removed and the cells were washed and fixed with 20% isopropanol and 10% formaldehyde, respectively. The fixed 3T3-L1 cells were stained with ORO working solution for 10 min. Subsequently, the cells were washed with water. The absorbance of the ORO extracted by isopropanol was measured at 520 nm by a spectrophotometer.

### Triglyceride (TG) deposition in 3T3-L1 cells

The protocols of 3T3-L1 cell differentiation and treatment with α-tocopherol, LA, or CLA were the same as for the ORO assay. The TG accumulation in the cells (nmol/µg protein) was measured with a TG colorimetric kit (Biovision), according to the manufacturer’s instructions.

### Cellular uptake of the nanoparticles

To evaluate the nanoparticle uptake by 3T3-L1 cells, free CLA or CLA-loaded NLCs were incubated with the cells (1 × 10^5^ cells/well) for 12, 24, and 48 h. The harvested cells were centrifugated at 500 ×*g* for 10 min, and then the cell pellet was lysed with lysis buffer (0.5 ml/well). The intracellular CLA content was quantified by LC/MS. The nanoparticles were also labeled with fluorescence dye (10 µg/ml rhodamine 800) to detect nanoparticle internalization in cells by flow cytometry and confocal microscopy. After incubation of the cells with the dye-loaded nanoparticles for 24 h, fluorescence from a gated population of 3T3-L1 cells (10,000 cells) was acquired on channel FL3, with excitation at a wavelength of 488 nm. The cell uptake of nanoparticles was also monitored by confocal microscopy (TCS SP8 X AOBS, Leica). The adipocytes were stained with 4’,6-diamidino-2-phenylindole (DAPI) and LysoTracker (Invitrogen) to observe the nuclei and lysosomes, respectively.

### Western blotting

Immunoblotting was performed using differentiated 3T3-L1 cells after treatment with free or nanoparticulate CLA for 24 h. The 3T3-L1 cells were washed twice with cold PBS, and lysed with lysis buffer for 30 min. The cell lysate was centrifugated at 12,000 rpm for 20 min, and the supernatant was collected for immunoblotting analysis. The protein concentration in the supernatant was quantified by a Bradford assay (Bio-Rad). Equal amounts of proteins were denatured and separated by gel electrophoresis, and transferred to a nitrocellulose membrane. The blots were blocked in PBS containing 5% skim milk and 0.5% Tween 20 for 1 h, and then incubated with primary antibody (1:1,000) for 12 h and horseradish peroxidase-conjugated secondary antibody (1:5,000) for 1 h at room temperature. The primary antibodies were adipogenic transcription factors peroxisome proliferator activated receptor (PPAR)γ and CCAAT/enhancer-binding protein (C/EBP)α, lipogenic enzymes acetyl-CoA carboxylase (ACC) and fatty acid synthase (FAS). The immunoreactive protein concentrations were determined using an enhanced chemiluminescence kit (PerkinElmer). The concentrations of the proteins was calculated by the ratio of the densitometric measurement of the indicated protein to that of β-actin.

### Enzyme-linked immunosorbent assay (ELISA)

ELISA kits (BioLegend) were used to measure the adipokines produced by 3T3-L1 cells when inhibited by free or nanoparticulate α-tocopherol and CLA, following the manufacturer’s instruction. The levels of tumor necrosis factor (TNF)-α, interleukin (IL)-1β, IL-6, and leptin were measured, and the concentrations were determined based on a standard calibration curve.

### Animals

Sprague Dawley rats (5-week-old females) were obtained from BioLASCO, and maintained in a humidity- and temperature-controlled room (55% at 25 °C). All animals were treated in compliance with European Council Directive 86/609/EEC. The Institutional Animal Care and Use Committee of Chang Gung University approved the experimental protocol.

### In vivo treatment of rats with tocol NLCs

The rats were divided into 4 groups (6 rats for each group): (i) control (CTL): healthy rats were fed a standard diet (#5001, LabDiet) for 8 weeks; (ii) HFD: the rats were fed a HFD (D12492, Research Diets) for 8 weeks, PBS was intraperitoneally injected into the rats 2 times/week for the last 3 weeks; (iii) HFD + free form: the rats were fed a HFD (D12492, Research Diets) for 8 weeks, and free α-tocopherol (40 mg/kg) and CLA (4 mg/kg) in dimethyl sulfoxide (DMSO) was intraperitoneally injected into the rats 2 times/week for the last 3 weeks; and (iv) HFD + tocol NLCs: the rats were fed a HFD (D12492, Research Diets) for 8 weeks, and tocol NLCs containing α-tocopherol (40 mg/kg) and CLA (4 mg/kg) were intraperitoneally injected into the rats 2 times/week for the last 3 weeks. The rats were weighed twice per week. After 8 weeks of treatment, the rats were sacrificed to collect plasma and organs for analysis.

### Plasma biochemical analysis

Blood was collected from the rats, and plasma was obtained by centrifugation at 12,000 rpm at 4 °C for 15 min. Total cholesterol in plasma was detected using a Dimension EXL2000 Integrated Chemistry analyzer (Siemens), based on manufacturer’s instruction. The levels of aspartate aminotransferase (AST), alanine aminotransferase (ALT), blood urea nitrogen (BUN), and creatinine (CRE) were measured using laboratory kits (DRI-CHEM Slide, Fujifilm), in the automated analyzer (DRI-CHEM 4000i, Fujifilm).

### TG and adipokine analysis of organs/tissues

The expressions of TNF-α, IL-1β, and IL-6 in kidney, liver, spleen, groin, and epididymis were determined using ELISA kits. After weighing, the minced organs/tissues were added to PBS as the extraction medium, and homogenization using a MagNA Lyser (Roche). The supernatant of the homogenates was obtained by centrifugation at 12,000 rpm at 4 °C for 10 min. The detection protocols for TG and adipokines were the same as those used in the aforementioned cell-based studies.

### Histological examination

The excised organs/tissues were fixed in 10% formaldehyde, embedded in paraffin wax, and sliced at 5 μm for hematoxylin and eosin (H&E) staining. Liver tissues were also prepared for immunohistochemistry (IHC) analysis of FAS and F4/80. The sections were incubated with anti-FAS or anti-F4/80 antibody (1:500), and then incubated with biotinylated donkey anti-rabbit IgG. Photomicrographs of the slices were obtained using an optical microscope (DMi8, Leica).

### Nanoparticle biodistribution in rats

The tocol NLCs were labeled with DiR iodide (Thermo Fisher), a near-infrared (NIR) dye, to trace the nanoparticle biodistribution in vivo. The rats were anesthetized by intraperitoneal zoletil (25 mg/kg) and xylazine (6 mg/kg). The NLCs were administered to the rats by intraperitoneal injection. The real-time distribution of the nanocarriers was monitored using a Pearl Impulse Imaging System (Li-Cor) at 0, 1 min, 2 h, 4 h, 8 h, and 12 h after administration. The rats were then euthanized, and the organs/tissues were excised and rinsed with saline. The NIR signal in the organs/tissues was quantified using the in vivo imaging system.

### In vivo safety assay of the tocol NLCs

Eight-week-old healthy rats fed a normal diet were used for this experiment. The animals were divided into 3 groups (6 animals in each group): (i) CTL: the rats were intraperitoneally injected with saline 2 times/week for 3 weeks; (ii) free form: the rats were intraperitoneally injected with free α-tocopherol (40 mg/kg) and CLA (4 mg/kg) in DMSO 2 times/week for 3 weeks; and (iii) tocol NLCs: the rats were intraperitoneally injected with tocol NLCs containing α-tocopherol (40 mg/kg) and CLA (4 mg/kg) 2 times/week for 3 weeks. All rats were weighed twice per week for 3 weeks. At the end of the experiment, the animals were sacrificed to collect blood and organs/tissues for analysis of total cholesterol, biochemical parameters, and histology.

### Statistical analysis

Data were reported as the mean and standard error of the mean (SEM). The Kruskal-Wallis test was used to assess the significance of variations among the treatment groups, and Dunn’s test was used as a post hoc test to evaluate specific differences. Statistical significance was determined at the 0.05, 0.01, and 0.001 probability levels.

## Results

### Physicochemical characterization of tocol NLCs

The tocol NLCs were fabricated by heating, and then subjected to homogenization and sonication to produce nano-sized particles. To understand the effect of particle size on adipogenesis inhibition, NLCs of different sizes were prepared depending on the HP content. There was a gradual increase in the particle diameters with increasing amounts of HP (Table 1). The mean hydrodynamic size of small, medium, and large NLCs was 121, 137, and 151 nm, respectively. The size distribution of all nano-formulations was mono-modular, and narrow (< 0.3), suggesting an appropriate and uniform distribution in nanoscale. The nanocarriers showed a highly negative surface charge, with the smaller-sized NLCs displaying a greater negative zeta potential. Under the TEM, the three nanoformulations generally exhibited a round or oval shape (Supplemental Fig. 1). The diverse size distribution of the nanoparticles was significant for each nanoformulation. Basically, the size calculated from TEM confirmed the successful production of small, medium, and large nanocarriers. The diameter of NLCs estimated from TEM was lower than that of hydrodynamic size. This was due to the drying process for TEM observation, resulting in the shrinkage of nanoparticles. The encapsulation of CLA inside the nanoparticles was nearly 100% for all nano-formulations. The high encapsulation rate could be due to the lipophilic feature of CLA for loading in the lipid core. The storage stability of the tocol NLCs was examined for a period of 21 days. No significant change was found in sizes of the small, medium, or large NLCs after incubation in room temperature for 21 days (Supplemental Fig. 2).

**Table 1:** The characterization of tocol NLCs by particulate size, polydispersity index (PDI), zeta potential, and CLA encapsulation percentage

Formulation	Size (nm)	PDI	Zeta potential (mV)	CLA encapsulation (%)
Tocol NLCs (S)	120.53 ± 0.75	0.25 ± 0.02	-46.13 ± 2.62	96.32 ± 2.08
Tocol NLCs (M)	137.37 ± 0.48	0.24 ± 0.01	-43.77 ± 0.55	98.23 ± 3.40
Tocol NLCs (L)	151.43 ± 1.14	0.23 ± 0.01	-39.63 ± 0.32	99.59 ± 5.68

PDI, polydispersity index

Each value represents the mean ± SEM (*n* = 3)

### α-Tocopherol and CLA attenuate adipogenesis in 3T3-L1 cells

**Fig. 1:**
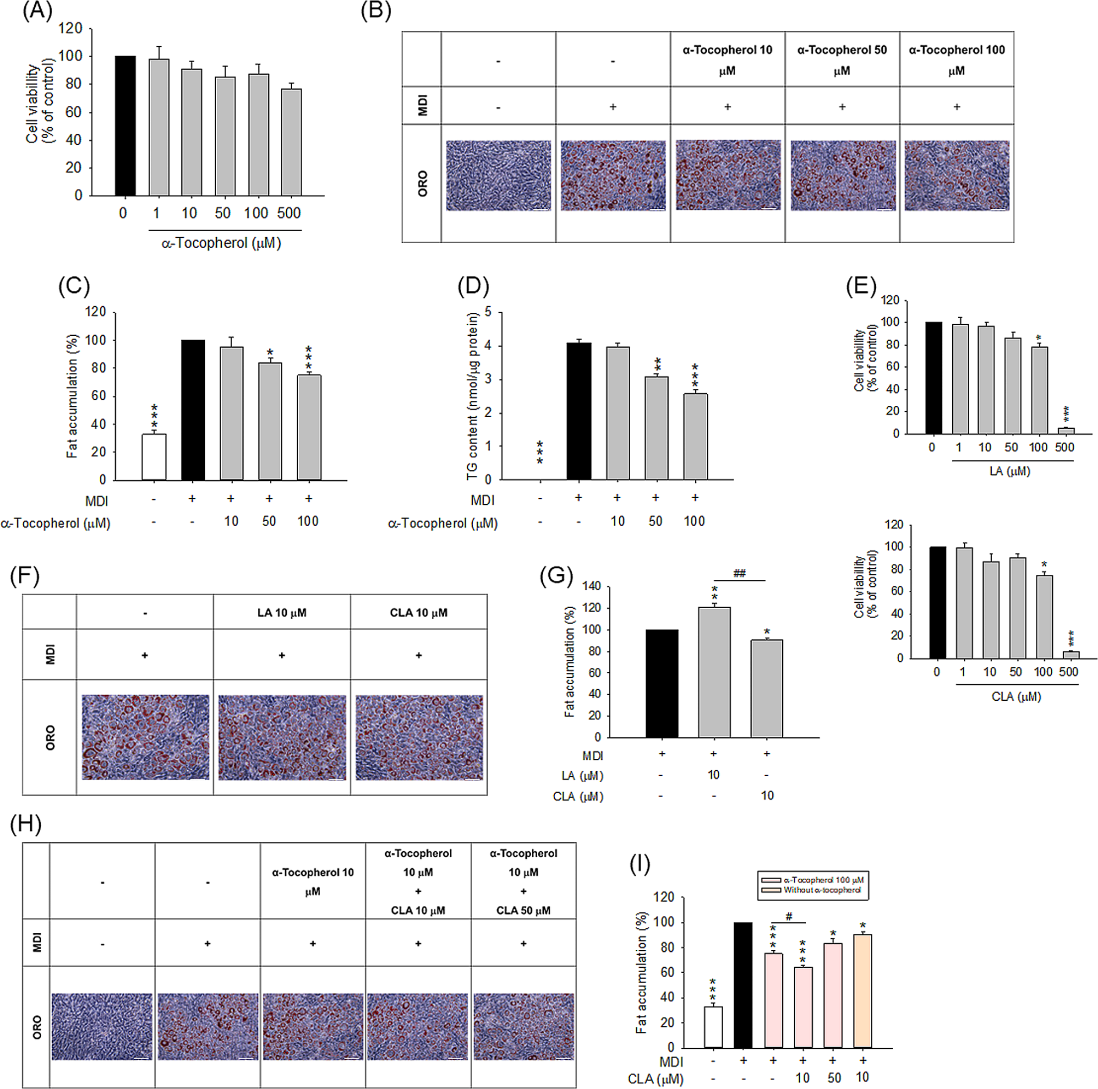
The 3T3-L1 cell viability and fat accumulation after treatment of α-tocopherol, LA, and CLA: (**A**) the cell viability after treatment of α-tocopherol as determined by MTT assay; (**B**) the ORO staining of 3T3-L1 cells after treatment of α-tocopherol with MDI stimulation; (**C**) the ORO quantification of 3T3-LI cells after treatment of α-tocopherol with MDI stimulation; (**D**) the intracellular TG content of 3T3-LI cells after treatment of α-tocopherol with MDI stimulation; (**E**) the cell viability after treatment of LA and CLA as determined by MTT assay; (**F**) the ORO staining of 3T3-L1 cells after treatment of LA and CLA with MDI stimulation; (**G**) the ORO quantification of 3T3-LI cells after treatment of LA and CLA with MDI stimulation; (**H**) the ORO staining of 3T3-L1 cells after treatment of combined α-tocopherol and CLA with MDI stimulation; and (**I**) the ORO quantification of 3T3-LI cells after treatment of combined α-tocopherol and CLA with MDI stimulation. All data are presented as the mean of three experiments ± S.E.M. *** *p* < 0.001; ** *p* < 0.01; * *p* < 0.05 as compared to the control group or MDI-treatment group. ^##^*p* < 0.01, ^#^*p* < 0.05 between the different treatment groups. Scale bar = 100 μm

To evaluate the anti-adipogenic activity of α-tocopherol, the effect of the free form of α-tocopherol on 3T3-L1 cell viability was evaluated first. The cell viability of > 80% is generally regarded as biocompatibility of the text compounds. α-Tocopherol at doses of ≤ 100 µM showed the viability of > 80% (Fig. 1A). The 3T3-L1 cells were differentiated into adipocytes for 8 days in the presence of free α-tocopherol, and then stained with ORO for determination of intracellular lipids. The microscopic appearance of the cells indicated the absence of lipid droplets in the undifferentiated cells (Fig. 1B). The induction of differentiation by the adipogenic cocktail resulted in lipid accumulation in the form of lipid droplets. The round morphology and the abundant lipid droplets simulated the metabolic features of the mature adipocyte phenotype. In contrast, treatment with α-tocopherol decreased ORO staining in a dose-dependent manner. Next, fat accumulation in 3T3-L1 cells was quantified and the results showed that α-tocopherol suppressed lipid accumulation in a concentration-dependent manner (Fig. 1C). Lipid deposition was decreased by 16% and 25% by treatment with 50 and 100 µM α-tocopherol, respectively. Notably, a concentration of 10 µM α-tocopherol did not influence lipid accumulation in the cells.

To verify the ORO staining results, intracellular TG content of 3T3-L1 cells was measured. The TG level in the differentiated adipocytes was increased as compared to the undifferentiated cells (Fig. 1D). The TG concentration in the undifferentiated pre-adipocytes was negligible. Similar to the ORO staining results, the TG level in α-tocopherol-treated cells was decreased at a 50 µM and 100 µM α-tocopherol concentration, but not at a 10 µM concentration. We next examined the anti-adipogenic effect of CLA and its isomer LA. Both free LA and CLA had no cytotoxic effects within the concentration range of 1–50 µM (Fig. 1E). Cells treated with 100 µM of LA and CLA exhibited a viability of 78% and 75%, respectively. Treatment with a concentration of 500 µM decreased cell viability to < 10%. Based on these data, a concentration of 10 µM was used for subsequent ORO staining. Free CLA at a concentration of 10 µM slightly but significantly decreased fat accumulation in 3T3-L1 adipocytes (Fig. 1F and G). Contrary to this result, LA at a concentration of 10 µM increased lipid deposition in the cells. Next, α-tocopherol (100 µM) and CLA (10 and 50 µM) were combined for testing the possible synergism of fat deposition inhibition. α-Tocopherol at 500 µM was ignored in the further experiments because of the cell viability of < 80% (76%). The combination of 100 µM α-tocopherol and 10 µM CLA resulted in a further reduction in lipid deposition, indicating a synergistic effect against adipogenesis (Fig. 1H and I). This effect was not detected with a CLA concentration of 50 µM.

#### The facile uptake of tocol NLCs in 3T3-L1 cells for enhancing anti-adipogenic activity

**Fig. 2:**
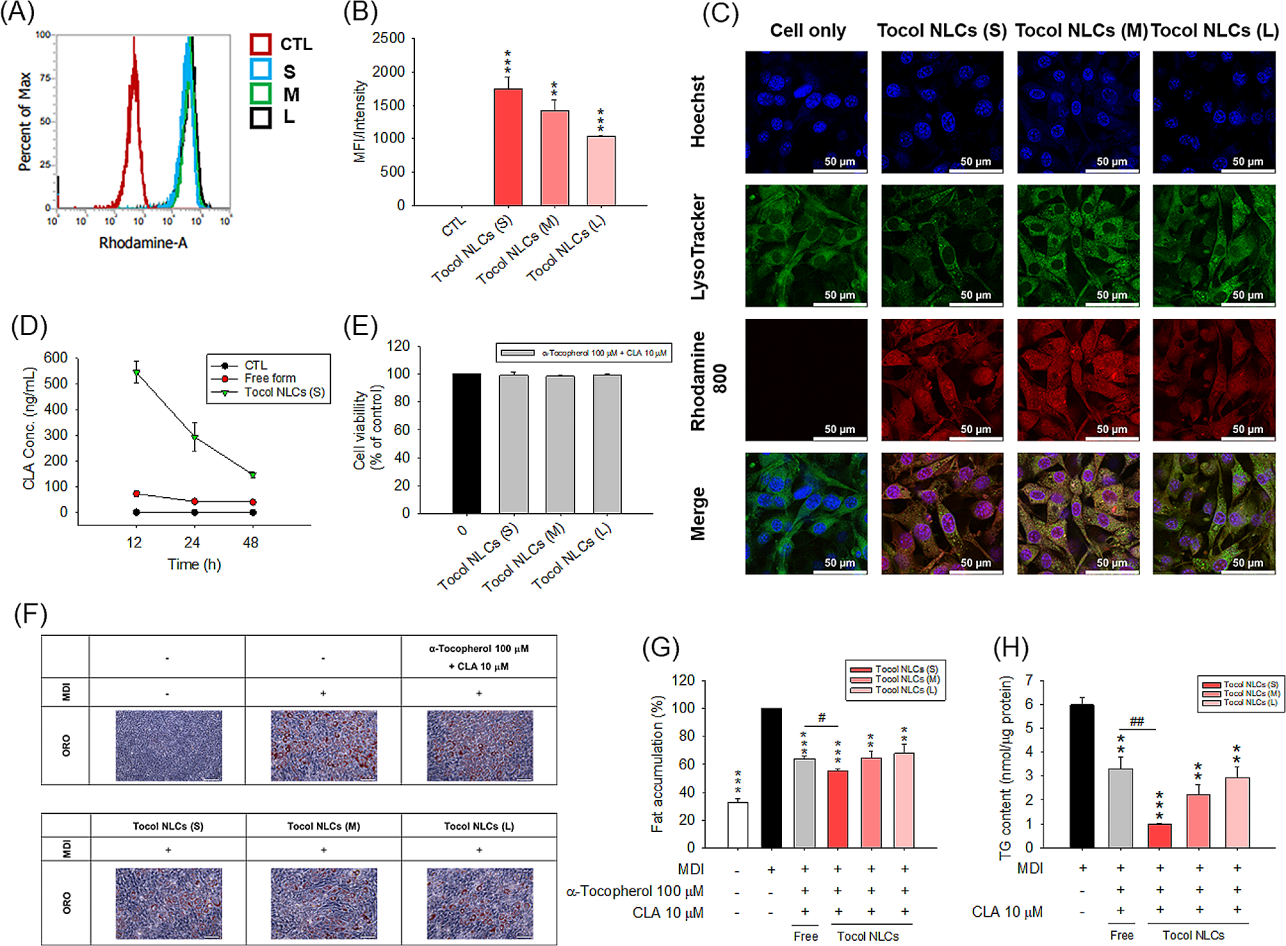
The 3T3-L1 cell uptake and fat accumulation after treatment of CLA-loaded tocol NLCs with different sizes: (**A**) flow cytometry analysis of CLA-loaded tocol NLCs incubated with 3T3-L1 cells; (**B**) the quantification of intracellular uptake of CLA-loaded tocol NLCs calibrated by the fluorescence intensity of the nanoformulation itself; (**C**) Confocal microscopic images of CLA-loaded tocol NLCs (red), DAPI (blue), and LysoTracker (green) showing uptake of nanoparticles by 3T3-L1 cells (Scale bar = 50 μm); (**D**) intracellular CLA uptake by 3T3-L1 cells after treatment with CLA-loaded tocol NLCs and free form; (**E**) the cell viability after treatment of CLA-loaded tocol NLCs as determined by MTT assay; (**F**) the ORO staining of 3T3-L1 cells after treatment of CLA-loaded tocol NLCs with MDI stimulation (Scale bar = 100 μm); (**G**) the ORO quantification of 3T3-LI cells after treatment of CLA-loaded tocol NLCs with MDI stimulation; and (**H**) the intracellular TG content of 3T3-LI cells after treatment of CLA-loaded tocol NLCs with MDI stimulation. All data are presented as the mean of three experiments ± S.E.M. *** *p* < 0.001; ** *p* < 0.01 as compared to the control group or MDI-treatment group. ^##^*p* < 0.01, ^#^*p* < 0.05 between the free and nanoparticulate forms

We investigated the effect of different sizes of NLCs on the cellular uptake and anti-adipogenesis effect of tocol nanocarriers. The NLCs were loaded with rhodamine 800, which emits red fluorescence, to estimate the cellular uptake of the nanoparticles. Flow cytometry data were recorded after incubation for 24 h. A remarkable increase in red fluorescence was found after incubation of 3T3-L1 cells with the nanoparticles (Fig. 2A). The fluorescence intensity of the cells was approximated for the 3 nano-formulations tested. Because different nano-formulations loaded with the fluorescence dye might display different fluorescence signals in the nanoparticles, we calibrated the mean fluorescence intensity (MFI) of the nanoparticle-treated cells by the fluorescence intensity of the nanoparticles themselves to reasonably compare the cell uptake of different sized NLCs. The results showed that nanoparticle uptake increased as the particle diameter decreased (Fig. 2B). Nanoparticle uptake was visualized using confocal microscopy. The control 3T3-L1 cells without NLC exposure did not exhibit rhodamine 800 fluorescence (Fig. 2C), while rhodamine 800 fluorescence was seen with tocol NLCs. Treatment with small and medium sized NLCs produced greater intracellular red fluorescence than treatment with large NLCs. The NLCs were co-located with lysosomes, suggesting lysosomal capture of the nanoparticles.

Since the small NLCs exhibited the greatest uptake by 3T3-L1 cells, they were selected to determine the intracellular CLA concentration. LC/MS analysis confirmed successful delivery of CLA into the adipocytes via small tocol NLCs (Fig. 2D). CLA inclusion in the nanoparticles increased the intracellular CLA delivery by 5.5-fold as compared to free form after a 12-h incubation. The CLA concentration in the NLC-treated cells decreased with an increase of treatment time. This could be due to CLA degradation in the cells, and the instability of NLCs after a long incubation time in the cell medium. No cytotoxicity was observed in cells treated with NLCs containing α-tocopherol (100 µM) and CLA (10 µM) (Fig. 2E), indicating cytocompatibility of the nanocarriers. We also found an increased cytocompatibility of CLA and α-tocopherol after nanoparticle incorporation. The avoidance of direct contact between free compounds and the cells by the nanocarriers had reduced the possible cytotoxicity elicited by the compounds themselves. Fat accumulation in 3T3-L1 adipocytes was reduced by tocol NLCs in a size-dependent manner, with the greatest reduction seen with the small NLCs (Fig. 2F and G). Small NLCs decreased lipid deposition by 45% as compared to the MDI-treatment group. This inhibition was significantly greater than that of combined α-tocopherol and CLA in free form (36%). The inhibition levels produced by medium and large NLCs was comparable to that of the free form. When small NLCs were added to MDI-treated adipocytes, the TG formed in the differentiated cells was reduced from 6 to 1 nmol/µg protein (Fig. 2H). This suppression was 3-fold greater than that produced by free α-tocopherol combined with CLA. In addition, small tocol NLCs without CLA did not prevent lipid accumulation in the differentiated 3T3-L1 cells (Supplemental Fig. 3). This indicate the importance of combining α-tocopherol and CLA in the nanocarriers to produce a synergistic effect.

### Tocol NLCs block transcription factors and lipogenic enzymes in adipocyte differentiation

**Fig. 3:**
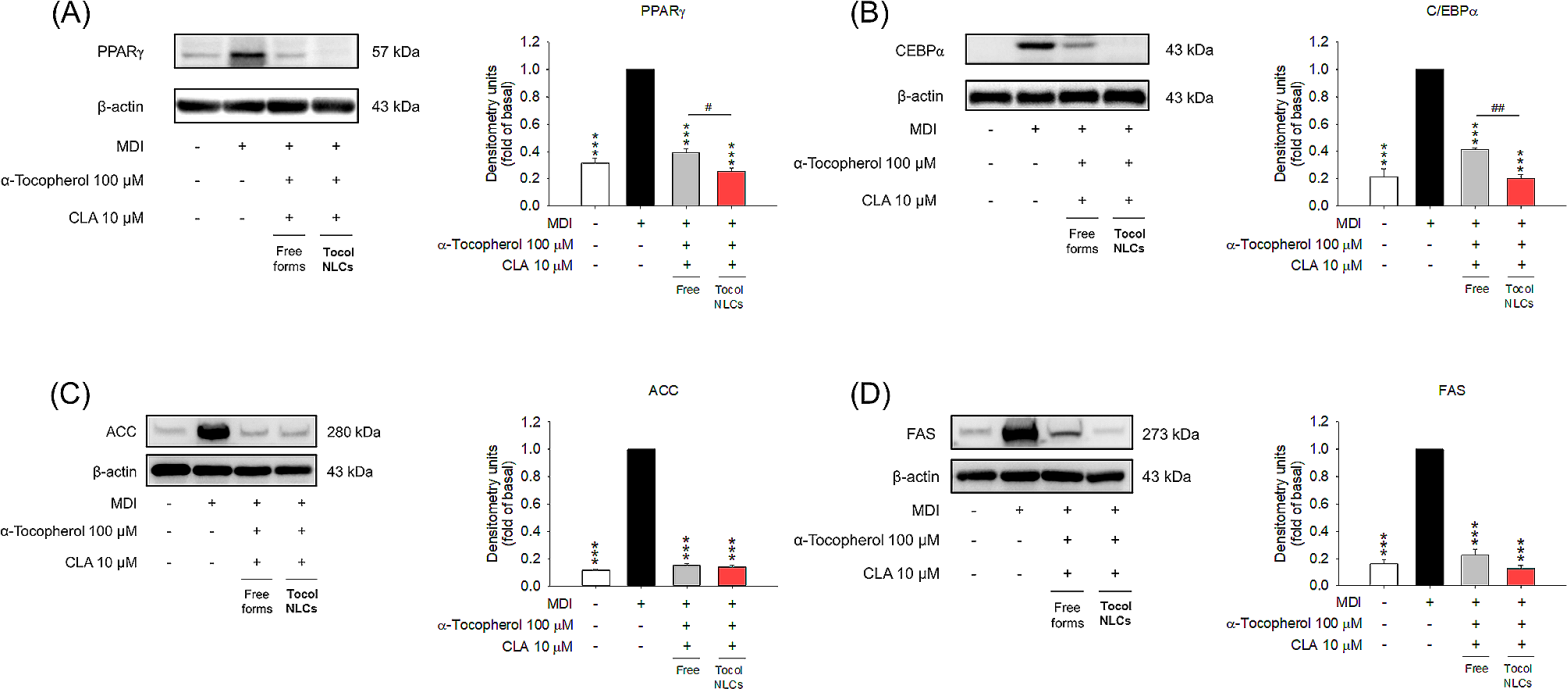
The effect of combined α-tocopherol and CLA in free or nanoparticulate form on transcription factors and lipogenic enzymes of 3T3-L1 cells: (**A**) the protein expression of PPARγ after treatment of combined α-tocopherol and CLA in free or nanoparticulate form; (**B**) the protein expression of C/EBPα after treatment of combined α-tocopherol and CLA in free or nanoparticulate form; (**C**) the protein expression of ACC after treatment of combined α-tocopherol and CLA in free or nanoparticulate form; and (**D**) the protein expression of FAS after treatment of combined α-tocopherol and CLA in free or nanoparticulate form. All data are presented as the mean of three experiments ± S.E.M. *** *p* < 0.001 as compared to the MDI-treatment group. ^##^*p* < 0.01, ^#^*p* < 0.05 between the free and nanoparticulate forms

To further explore the effect of CLA-loaded tocol nanocarriers on adipocyte differentiation, we estimated the protein levels of the transcription factors PPARγ and C/EBPα, and lipogenic markers ACC and FAS in 3T3-L1 cells with Western blotting. The transcription factors PPARγ and C/EBPα were significantly increased in MDI-treated cells (Fig. 3A and B, the uncut images of immunoblotting for triplicates are shown in Supplemental Fig. 4A and 4B). The densitometric quantification demonstrated a 3–5-fold increase in the level of transcription factors after MDI stimulation. Treatment of α-tocopherol and CLA in free or nanoparticulate forms blunted this upregulation, with the NLC forms showing greater suppression than the free form. The tocol NLCs decreased the levels of PPARγ and C/EBPα to the level of the control baseline. Treatment with MDI significantly increased the expression of ACC and FAS (Fig. 3C and D, the uncut images of immunoblotting for triplicates are shown in Supplemental Fig. 4C and 4D). Treatment of α-tocopherol and CLA in free or nanoparticulate forms resulted in a significant decrease in the expression of ACC and FAS.

We also examined FAS expression in adipocytes treated with Poloxamer 188, SPC, and NLCs without CLA to understand if chemicals in NLCs could mitigate lipogenesis. No FAS inhibition was detected when adipocytes were treated with Poloxamer 188 and SPC, suggesting that α-tocopherol and CLA were the major ingredients in the nanocarriers responsible for the anti-lipogenic activity (Supplemental Fig. 5). The results suggest that the combination of α-tocopherol with CLA in NLCs is essential for blocking lipogenesis, since the tocol nanosystems without CLA failed to downregulate FAS.

#### Tocol NLCs inhibit adipokine overexpression in adipocyte differentiation

**Fig. 4:**
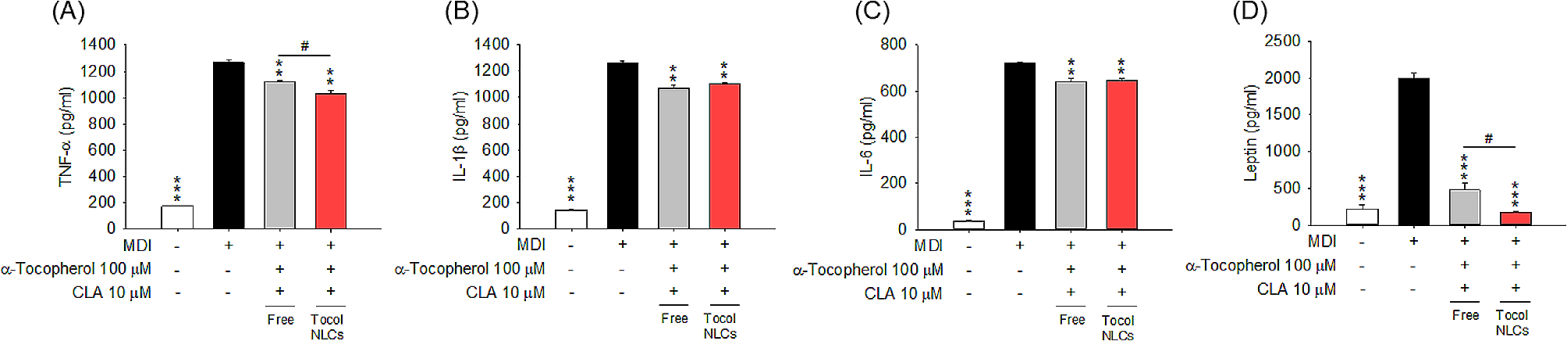
The effect of combined α-tocopherol and CLA in free or nanoparticulate form on adipokines of 3T3-L1 cells: (**A**) TNF-α; (**B**) IL-1β; (**C**) IL-6; and (**D**) leptin. All data are presented as the mean of three experiments ± S.E.M. *** *p* < 0.001; ** *p* < 0.01 as compared to the MDI-treatment group. ^#^*p* < 0.05 between the free and nanoparticulate forms

We measured changes in the protein levels of adipokines in MDI-treated 3T3-L1 cells treated with combined α-tocopherol and CLA. The adipokines tested were TNF-α, IL-1β, IL-6, and leptin. MDI intervention greatly upregulated the expressions of the adipokines in the cells (Fig. 4). Treatment of tocol NLCs reduced TNF-α expression by 19%, compared to 12% in the free form treatment group (Fig. 4A). Comparable inhibition of IL-1β and IL-6 in the free and nanoparticulate compounds was seen (Fig. 4B and C). Leptin upregulation by MDI was blocked by combined α-tocopherol and CLA in the free or nanoparticulate form (Fig. 4D), notably NLCs. CLA-loaded tocol nanocarriers reduced leptin expression by 92%, a level that was approximately the same as the control baseline value.

#### Tocol NLCs mitigate adipogenesis in obese rats

**Fig. 5:**
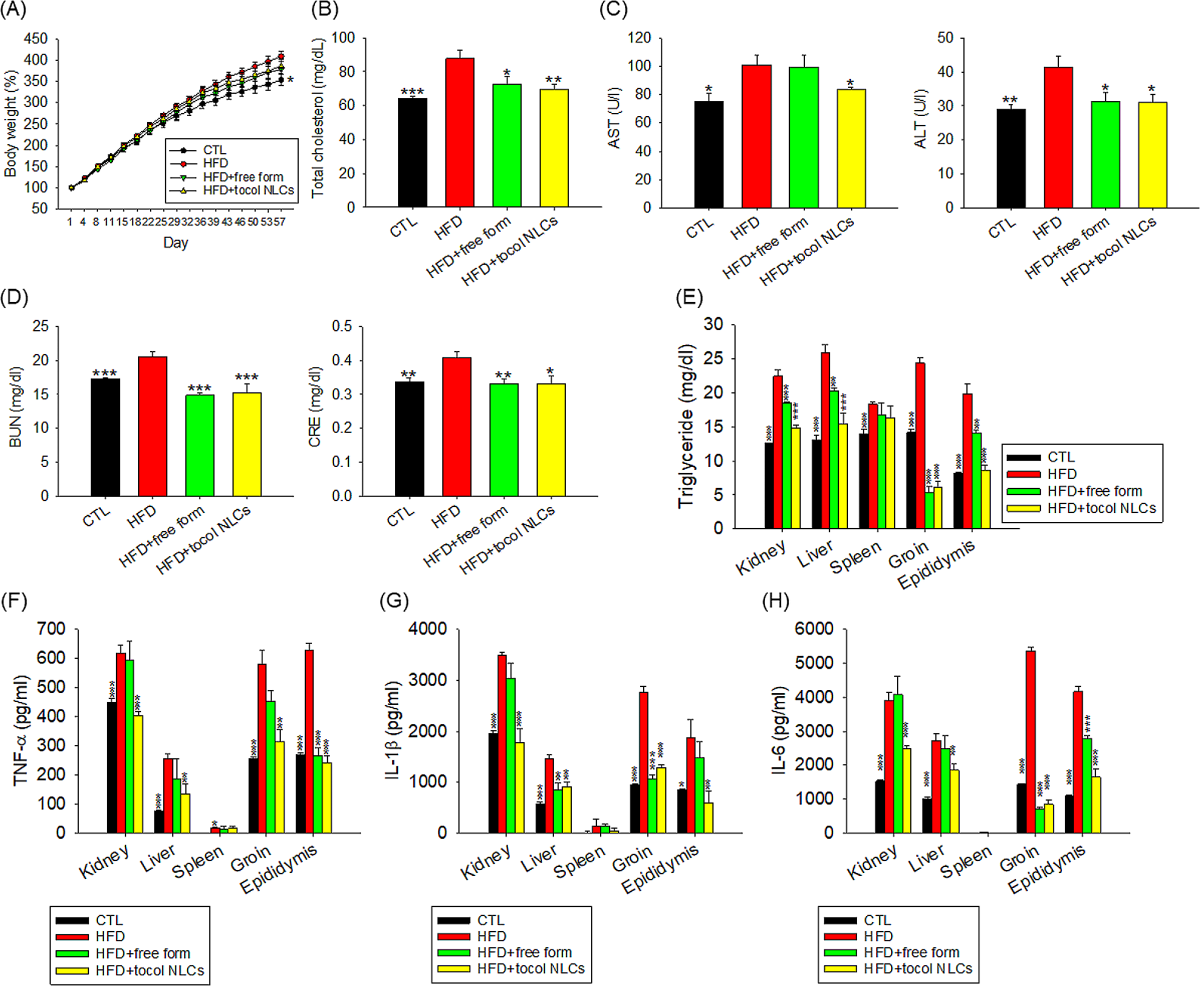
The antiobesity effect of combined α-tocopherol and CLA in free or nanoparticulate form by intraperitoneal administration on the HFD-fed obese rats: (**A**) the body weight change as compared to normal diet group during the HFD-fed period; (**B**) total cholesterol in plasma after HFD consumption for 8 weeks; (**C**) AST and ALT concentration in plasma after HFD consumption for 8 weeks; (**D**) BUN and CRE concentration in plasma after HFD consumption for 8 weeks; (**E**) TG in organs/tissues after HFD consumption for 8 weeks; (**F**) TNF-α in organs/tissues after HFD consumption for 8 weeks; (**G**) IL-1β in organs/tissues after HFD consumption for 8 weeks; and (**H**) IL-6 in organs/tissues after HFD consumption for 8 weeks. All data are presented as the mean of six experiments ± S.E.M. *** *p* < 0.001; ** *p* < 0.01; * *p* < 0.05 as compared to the HFD intervention alone

Because the small tocol NLCs were highly internalized in adipocytes, and caused decreased adipogenesis in vitro, we further investigated the anti-obesity effect of the nanocarriers using a HFD rat obesity model. Rats fed with a HFD simulates the consequences of a Western diet in humans. The administration of small tocol NLCs by intraperitoneal injection was performed to minimize the off-target effects to adipose tissues. The body weight of the rats fed the HFD was significantly increased by 15% at the end of experiment (Fig. 5A). The HFD-induced weight gain was reduced by free and nanoparticulate α-tocopherol/CLA, with a similar reduction for both forms. Serum total cholesterol was significantly increased in the rats fed the HFD, and the increase was significantly suppressed by free and nanoparticulate compounds, with a similar reduction for both forms (Fig. 5B). The HFD increased AST and ALT levels compared with the normal diet group (Fig. 5C). The CLA-loaded tocol nanocarriers inhibited the increase of AST in HFD rats, but inhibition of AST increase was not observed in the free α-tocopherol/CLA group. In the case of ALT, both free form and nanocarriers inhibited ALT increase in the HFD rats. A similar finding was noted for BUN and CRE (Fig. 5D). Rats treated with combined α-tocopherol and CLA had lower levels of BUN and CRE, which were approximately the same as that of healthy rats fed a normal diet.

To determine the lipid content in the abdominal cavity after intraperitoneal nanoparticle delivery, the amount of TG in the kidney, liver, spleen, groin, and epididymis was determined. As expected, the TG concentration in the peripheral organs of obese rats was increased compared to the healthy control (Fig. 5E). The increase of TG levels in all of the organs except the spleen was significantly inhibited by treatment with combined α-tocopherol and CLA. The NLCs attenuated TG increase in the kidney, liver, and epididymis with a lower level compared to the free form. The cytokines TNF-α, IL-1β, and IL-6 were all upregulated in the peripheral organs of HFD rats (Fig. 5F and H). The increased cytokine expression in the HFD rats was attenuated by NLCs, but not by the free form. The same result was observed for TNF-α and IL-6 in the liver. NLCs generally exhibited a superior suppression of the overexpressed cytokines compared to the free form in the adipose tissues (groin and epididymis) of the HFD rats. The expressions of the cytokines in the spleen were very low, and treatment with combined α-tocopherol and CLA did not significantly change the expressions of the cytokines.

#### The biodistribution of tocol NLCs and histological examination

**Fig. 6:**
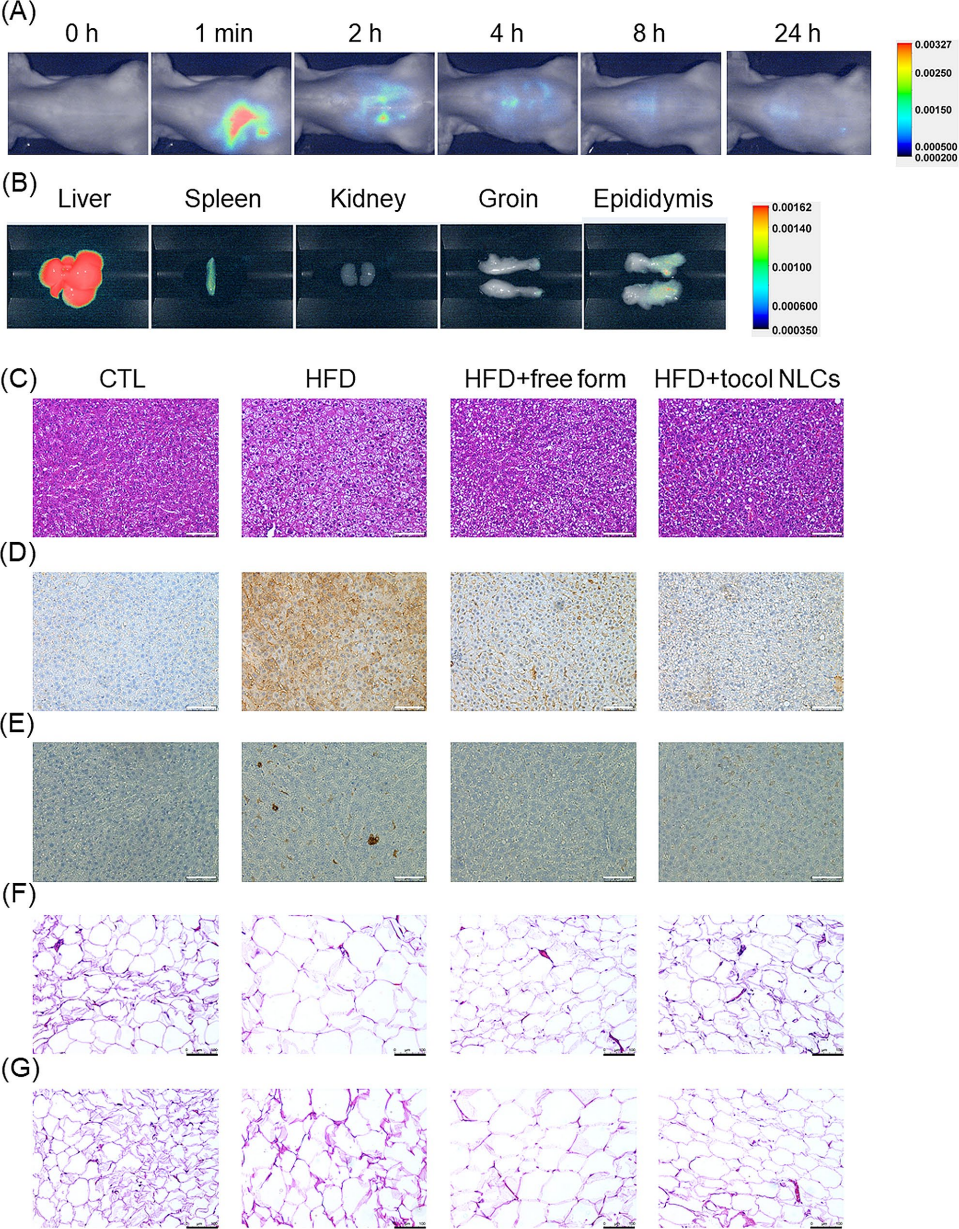
Biodistribution and histological examination of the HFD-fed obese rats after the intraperitoneal administration of combined α-tocopherol and CLA in free or nanoparticulate form: (**A**) real-time bioimaging of the CLA-loaded tocol NLCs in the rats within 24 h; (**B**) the biodistribution of CLA-loaded tocol NLCs in different organs/tissues at 24 h post-administration; (**C**) H&E staining of liver; (**D**) FAS expression of the liver determined by IHC; (**E**) F4/80 expression of the liver determined by IHC; (**F**) H&E staining of groin; and (G) H&E staining of epididymis. Scale bar = 100 μm

The tocol NLCs were labeled with DiR iodide, a NIR dye, to monitor their biodistribution using a bioimaging system. Real-time bioimaging showed that the nanocarriers remained at the injection site (abdominal cavity) after local administration (Fig. 6A). The NIR signal decreased with increasing time, indicating biodegradation of the nanoparticles. The peripheral organs in the abdominal cavity were retrieved 24 h after injection for ex vivo bioimaging. The results showed that when administered by intraperitoneal injection, the nanocarriers had a tendency to deposit in the liver (Fig. 6B). There were some weak NIR signals in the spleen, groin, and epididymis. The NIR signal in the organs was quantified at 4 h after injection. The greatest accumulation of NLCs was in the liver, followed by the epididymis and groin (Table 2).

**Table 2:** The NIR intensity of different organs after the i.p. administration of tocol NLCs for 24 h

Organ	NIR intensity
Liver	1322.60 ± 21.44
Spleen	30.88 ± 5.58
Kidney	5.49 ± 0.17
Groin	27.69 ± 5.36
Epididymis	43.46 ± 9.39

Because obesity is closely associated with increased fat in the liver and increased adipose tissue, we examined the histology of the liver, groin, and epididymis. The HE stained sections of the liver of rats fed a normal diet exhibited a normal arrangement of hepatocytes with intact morphology (Fig. 6C). A HFD significantly enriched the fat vacuoles in the liver tissue. The amount of fat deposition was diminished by treatment with free and nanoparticulate α-tocopherol/CLA. The reduction by the free form was almost the same as that of tocol NLCs. The IHC staining for FAS showed a large deposition in the liver of HFD rats (Fig. 6D). Free α-tocopherol combined with CLA significantly reduced FAS overexpression in the HFD rats. A further reduction was observed after treatment with α-tocopherol and CLA loaded into NLCs. Macrophage infiltration in the liver was observed by F4/80-labeled IHC. Some macrophages were recruited into the liver in the HFD rats (Fig. 6E). Combined α-tocopherol and CLA in free or nanoparticulate form hindered this accumulation, indicating suppression of inflammation. The adipocyte size of the groin in HFD rats was remarkedly enlarged, (Fig. 6F), demonstrating cell differentiation and lipid accumulation in the adipocytes. This effect was reduced by treatment with free form and NLCs. Adipocyte hypertrophy was also observed in the epididymis of HFD rats (Fig. 6G). The adipocyte size did not show difference between the HFD group and free form-treated group. The adipocyte size in the epididymis in the nanocarrier-treated group was visually smaller as compared to that of HFD obese rats.

### The safety of tocol NLCs in healthy rats

**Fig. 7:**
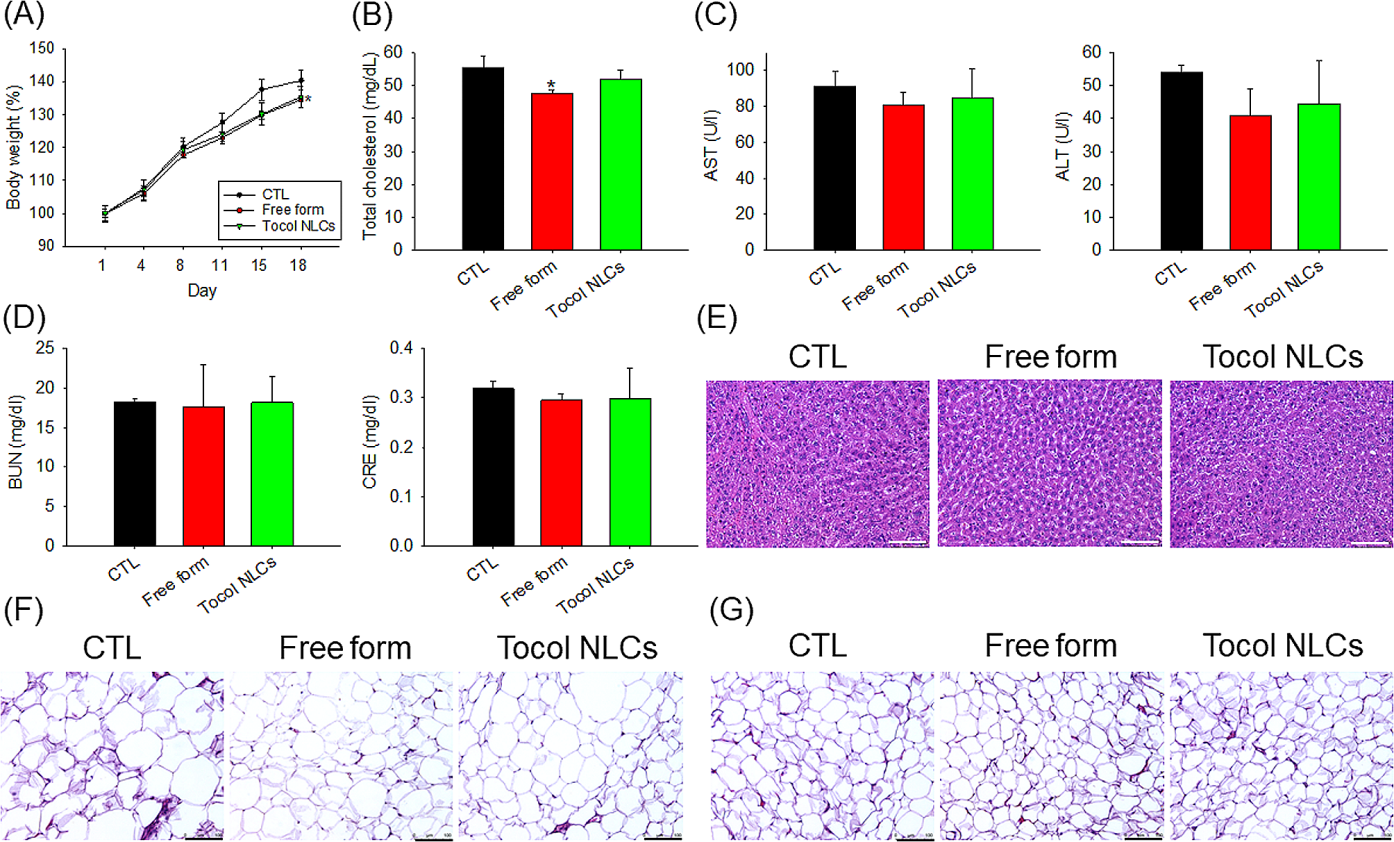
The safety assessment of combined α-tocopherol and CLA in free or nanoparticulate form by intraperitoneal administration on the healthy rats: (**A**) the body weight change during 3 weeks of repeated (6 times) administration; (**B**) total cholesterol in plasma after 3 weeks of repeated (6 times) administration; (**C**) AST and ALT concentration in plasma after 3 weeks of repeated (6 times) administration; (**D**) BUN and CRE concentration in plasma after 3 weeks of repeated (6 times) administration; (**E**) H&E staining of liver; (**F**) H&E staining of groin; and (**G**) H&E staining of epididymis. All data are presented as the mean of six experiments ± S.E.M. * *p* < 0.05 as compared to the control group. Scale bar = 100 μm

Finally, we examined the possible toxicity of the free and nanoparticulate α-tocopherol/CLA given by local administration. Three weeks of administration of combined α-tocopherol and CLA in healthy rats slightly reduced the body weight by about 4%, when both free and nanoparticulate forms were administered (Fig. 7A). The serum total cholesterol level was decreased by the free form, but not by NLCs (Fig. 7B). No significant changes were observed in hepatic and renal function indices between the sham control and α-tocopherol/CLA-treated rats (Fig. 7C and D). The histological analysis of organ sections (liver, groin, and epididymis) illustrated no observable lesions and no morphological changes after treatment with α-tocopherol/CLA.

## Discussion

Obesity is a worldwide health problem, and the prevalence is increasing rapidly. The present study investigated a nanoparticle delivery system composed of α-tocopherol and CLA for the inhibition of adipogenesis through targeting adipocytes and adipose tissues. The hybridization of liquid oil and solid lipid in the NLC core leads to imperfections of the crystalline matrix [21], contributing to the high entrapment of CLA molecules in NLCs. It has been reported that lipophilic molecules are generally more soluble in liquid lipid than solid lipid [22]. CLA exhibits high encapsulation in tocol NLCs because of the higher percentage of α-tocopherol than solid HP. The particle size of tocol NLCs remained unchanged during 21 days at room temperature; the solid lipid incorporation in NLCs enhances the stability of the nanoparticles [22]. Stable NLCs should possess a minimum zeta potential of ≥ |20| mV to exhibit sufficient electrostatic repulsion [23]. Our NLCs fit this criterion. The nanoparticulate surface decoration by Poloxamer surfactants also provides the bulky and steric repulsion against particle aggregation [24].

An increase of adipose tissue results from an increase of adipocyte number and lipid droplet size [25]. We found that free α-tocopherol and CLA reduced the ORO-stained lipid droplets in 3T3-L1 cells, and the combination of both compounds further decreased the level of lipid droplets. CLA has been reported to reduce adipocyte size in body fat mass [26]. It has also been reported that α-tocopherol induces the change of mature adipocytes to beige adipocytes, which are cells with a reduced lipid droplet size [27]. The differentiated adipocytes have the ability to store TG in lipid droplets [28]. The excessive TG deposition in the adipocytes evokes the development of obesity. Our data showed that free α-tocopherol inhibits TG accumulation.

It should be cautious to discuss the anti-adipogenic activity of free CLA, since fat accumulation of adipocytes was attenuated by a concentration of 10 µM, but not by 50 µM. LA, an isomer of CLA, at a concentration of 10 µM increased lipid accumulation. CLA is readily fused into TG in fat stores and phospholipids in cell membranes [11]. The presence of more lipids in the form of CLA contributes to the filling of lipid droplets [29]. LA is abundant in the Western diet, and induces increased lipid peroxidation [30]. It also interferes with TGs to increase the lipid content and size of adipocytes [31]. Thus, treatment with a higher concentration of CLA (50 µM) and LA was unfavorable for counteracting adipogenesis. The optimization of CLA dose for anti-adipogenesis therapy is critical. We selected a CLA concentration of 10 µM to prepare tocol NLCs. The flow cytometry and confocal microscopy experiments depicted a facile uptake of tocol NLCs in adipocytes. This internalization produced no cytotoxicity, suggesting cytocompatibility of the nanomaterials. The intracellular CLA amount was greater in the nanocarrier-treated cells compared to those treated with free CLA. Previous study [10] has shown that CLA in the free fatty acid form exhibits limited uptake by 3T3-L1 cells. Our data demonstrated that tocol NLCs could bind to adipocytes, and were largely internalized into the cells. The NLCs can be classified as soft nanoparticles, which are deformed in the process of cellular internalization [32]. In addition to anti-adipogenesis activity, α-tocopherol in the NLCs is beneficial for enhancing cellular uptake since this molecule greatly intercalates into the phospholipid bilayers of the cell membrane [17]. The coating of soy phospholipids on our nanoparticle surface also improved the affinity of the nanoparticles for the cell membrane.

The cellular uptake of the nanoparticles can be altered by modulating the particle size. Our data revealed that the lipid-based nanocarriers with the smallest size exhibited the greatest adipocyte internalization. The increased surface-to-mass ratio of the small size nanoparticles could enhance the frequency of interaction with adipocytes. Smaller-sized nanoparticles usually require less energy for cellular uptake [33]. Confocal microscopy showed the localization of the nanoparticles in intracellular lysosomes. After internalization into the cells, the lipids in the lipid-based nanoparticles ionized at low pH in lysosomes, enabling lysosomal escape and the release of the bioactive compounds into the cytoplasm [34]. This could be the pathway by which CLA-loaded tocol NLCs produce an anti-adipogenic effect. Positively charged nanoparticles often exhibit more favorable cellular ingestion than negatively charged nanoparticles because of the anionic proteoglycans in the cell membrane. Our nanocarriers had a negative surface charge, and in adipocytes there are some cationic sites in the cell membrane which facilitate the penetration of negatively charged nanoparticles upon binding [35].

Adipogenesis is a complicated process, that involves different signaling pathways and transcription factors. PPARγ and C/EBPα are the main adipogenesis-regulating transcription factors, and are important in the early steps of adipocyte differentiation [36]. PPARγ is a nuclear receptor that is responsible for lipid droplet formation in adipogenesis [37]. This receptor cooperates with C/EBPα to orchestrate adipocyte differentiation and inflammation. Both α-tocopherol and CLA can act as PPARγ/C/EBPα antagonists, as evidenced by our data. The combination of α-tocopherol and CLA in NLCs produced a greater decrease of transcription factor expression than the free form. It has been reported that *t*10-*c*12 CLA downregulates the transcription of PPARγ to prevent TG deposition in 3T3-L1 cells [38]. However, a recent study [39] demonstrated the activation of PPARγ by CLA. This discrepancy could be due to PPARγ upregulation induced by a high dose (100 µM) of CLA. The optimization of CLA dose is important for an anti-adipogenesis effect. Our results showed that a CLA concentration of 10 µM in tocol NLCs was useful to counteract obesity. The activation of PPARγ prompts the expression of lipogenic enzymes such as ACC and FAS [40]. Both proteins control the later stage of adipocyte differentiation, for regulating adipogenesis, glucose metabolism, and energy expenditure [41]. The immunoblotting assay suggested that tocol NLCs prevent lipid accumulation through blockade of lipogenic pathways. The lipid-based nanocarriers may act as an ACC/FAS inhibitor.

The dysfunctional adipocytes in obesity secret proinflammatory adipokines to recruit immune cells in adipose tissues [42]. The inflammation that occurs in adipose tissue due to the release of TNF-α, IL-1β, and IL-6 is a crucial factor in the development of cardiovascular disorders, diabetes, and cancers [41]. IL-1β upregulation in 3T3-L1 cells can increase the expression of TNF-α and IL-6, contributing to an increase of TG storage and body fat. Previous studies [12, 29] have demonstrated the anti-inflammatory activity of CLA, which blunts cytokine release in adipose tissues by inhibiting obesity-induced TNF-α and IL-6 [30, 43]. A clinical trial [44] also showed a synergistic anti-inflammatory activity of α-tocopherol and CLA in rheumatoid arthritis patients. Our ELISAs showed a reduction of TNF-α, IL-1β, and IL-6 overexpression in differentiated adipocytes after treatment with combined α-tocopherol and CLA. However, no significant difference between free and nanoparticulate forms was noted for IL-1β and IL-6 in the cell-based study. A stronger cytokine inhibition by NLCs than the free form was found in the animal-based study. This could be due to the protection of α-tocopherol and CLA by the nanocarriers to prolong the half-life of the compounds in the in vivo condition. The reduced biodegradation by NLCs was not observed in the in vitro condition due to the absence of metabolism by enzymes and proteins. Leptin is one of the adipokines with proinflammatory activity, and upregulates TNF-α and IL-6 expression [45, 46]. Leptin is also a target gene of PPARγ and C/EBPα. We found that the CLA-loaded tocol NLCs mitigated the overexpression of leptin in adipocytes to a greater degree as compared to the other cytokines.

Small-sized NLCs were intraperitoneally administered to HFD rats for evaluating the anti-obesity effect. The lipid-based nanocarriers were shown to accumulate in the liver and adipose tissues for effectively suppressing fat deposition and the inflammatory response. Hepatic and adipose tissues are the major lipogenic tissues in the body [47]. Most of the injected NLCs accumulated in the liver. The lipids released from adipose tissues in obesity are deposited in peripheral organs, such as the liver [48]. It is not surprising that the nanoparticles accumulate in the liver because the liver is the main metabolic site receiving xenobiotics. α-Tocopherol transfer protein (α-TTP) is a liver cytosolic transport protein that facilitates α-tocopherol uptake into the liver, and retains a high level of α-tocopherol in the liver [49]. The tocol NLCs might be recognized by α-TTP, facilitating nanoparticle transport into the liver. The liver is a vital organ in the maintenance of lipid and glucose homeostasis. The HFD rats demonstrated a higher TG reduction in the liver by nano-encapsulated α-tocopherol and CLA than the free form. The number of lipid vacuoles in the liver tissue were also decreased by NLCs. The transcription of ACC and FAS is increased in the liver of patients with fatty liver diseases [50]. The IHC and cell-based studies confirmed the ability of tocol NLCs to inhibit FAS overexpression. The adipokines are not only secreted from adipocytes, but also the resident cells of macrophages and endothelial cells in liver. Excessive fat deposition also promotes increased macrophage and neutrophil infiltration in the liver [51]. Adipokines play a key role in the interplay between adipocytes and macrophages in obesity-induced inflammation, and form a repetitive loop [52]. Consistent with our in vitro data, NLC administration to HFD rats resulted in reduced cytokine levels in the liver and adipose tissues. This could be due to the suppression of both adipocyte and macrophage activation, since macrophage recruitment into the liver was prevented by the nanocarriers.

Adipose tissue is responsible for storing most fat in the body, and adipocyte hypertrophy and hyperplasia leads to obesity and metabolic syndrome [41]. The body fat reduction by CLA is due to a decrease of adipocyte size rather than adipocyte number [26]. Our in vivo study showed shrinkage of hypertrophied adipocytes in the groin and epididymis of obese rats treated with CLA-loaded tocol NLCs. Adipose tissue is an energy reservoir, and also an immune tissue that is a primary source of cytokines [46]. Adipose tissue in obesity secrets proinflammatory mediators such as TNF-α, IL-1β, and IL-6 [52]. A reduction of adipocyte hypertrophy is directly associated with a decrease of IL-6 [53]. Our results showed that tocol NLCs counteracted cytokines in groin and epididymis with a lower or comparable level than free form. Suppression of adipokines is especially favorable for blocking chronic inflammation in the adipose tissue, preventing the associated metabolic syndrome. We did not determine leptin in the animal experiment. Leptin is a circulating cytokine [54]. We had detected leptin concentration in serum and found no significant difference between the groups receiving normal diet and HFD (0.23 versus 0.25 nmol/l). Previous study [55] demonstrates that leptin is even decreased in the rats receiving short-term HFD. Thus leptin might not be an ideal obesity indicator in this study.

It is important to establish the safety profile of nanoparticles prior to human studies. Previous study [38] has reported a toxicity in mice receiving the excess *t*10-*c*12 CLA. A CLA dose of 4 mg/kg in NLCs did not cause toxicity in the healthy rats, based on serum biochemical parameters and histological examination of tissues. We concluded that the CLA-loaded tocol nanocarriers given via local administration has the potential to protect against obesity with limited adverse effect. One of the special features of CLA-loaded tocol nanocarriers is the improved delivery of CLA to adipocytes and liver/adipose tissues with their passive targeting characteristics. The active targeting of the nanocarriers by ligand conjugation always causes a high cost. The tocol NLCs offer a low-cost, simple, targeted, and synergistic approaches for treating obesity. The tocol NLCs containing CLA have diverse benefits that are produced simultaneously: (i) satisfactory storage stability; (ii) facile uptake by adipocytes to inhibit fat accumulation, TG deposition, and adipokine overexpression; (iii) accumulation in the liver to reduce TG levels, promote adipokine upregulation, and lipid vacuoles; (iv) adipose tissue distribution to counteract adipocyte hypertrophy; and (v) an acceptable safety profile.

There are some limitations of this study. We used intraperitoneal administration to deliver the tocol NLCs for minimizing the off-target effect. Local administration could reduce undesired effects and the CLA dose required. Nevertheless, intraperitoneal injection is not a common method of administering medications. Further study is needed to explore the anti-adipogenic effect of the NLCs using the other administration routes. For instance, the subcutaneous delivery of the nanocarriers may be effective for treating subcutaneous adipose tissue. Another limitation is that no animal model can totally reflect the complexities of human obesity. The extrapolation of our data to clinical therapy is yet to be established. The other concern is that the NLCs did not reduce the adipogenesis to the baseline control based on TG and cytokine levels in vivo. The continuous optimization of the nanoformulation by modifying dose, treatment protocol, and administration route may be beneficial to maximize the bioactivity.

## Conclusions

In conclusion, the prepared NLCs were largely internalized in adipocytes, and this uptake of the nanocarriers increased CLA delivery by 5.5-fold. The tocol NLCs blocked adipocyte differentiation more profoundly as compared to a corresponding dose of free α-tocopherol and CLA. Compared with the combination of free α-tocopherol and CLA, NLCs led to a greater inhibition of fat accumulation, TG deposition, and adipokine expression in mature adipocytes. The NLCs significantly attenuated the overexpression of PPARγ and C/EBPα, the main adipogenic transcription factors, in the adipocytes. In addition, the levels of the lipogenic markers ACC and FAS were decreased by nanoparticle treatment. HFD consumption in rats produced obesity, adiposity, liver steatosis, and adipose tissue hypertrophy, and these were effectively suppressed by the CLA-loaded nanocarriers. The in vivo bioimaging illustrated the highest accumulation of the NLCs in the liver, followed by adipose tissues. Most of the currently used anti-obesity drugs have psychiatric and cardiovascular side effects. There is a space for the design of novel anti-obesity drugs or formulations. The nanosystems developed in this investigation may be an alternative for treating obesity.

### Electronic supplementary material

Below is the link to the electronic supplementary material.

Supplementary Material 1

## Data Availability

Data are available on request from the authors.
