# Supplementary material for: Facile adipocyte uptake and liver/adipose tissue delivery of conjugated linoleic acid-loaded tocol nanocarriers for a synergistic anti-adipogenesis effect
Source: J Nanobiotechnology. 2024 Feb 6;22:50. doi: 10.1186/s12951-024-02316-8 (PMC10845550; doi:10.1186/s12951-024-02316-8)
Supplement: Supplementary file 1 — Supplementary Material 1 [file 12951_2024_2316_MOESM1_ESM.pdf]

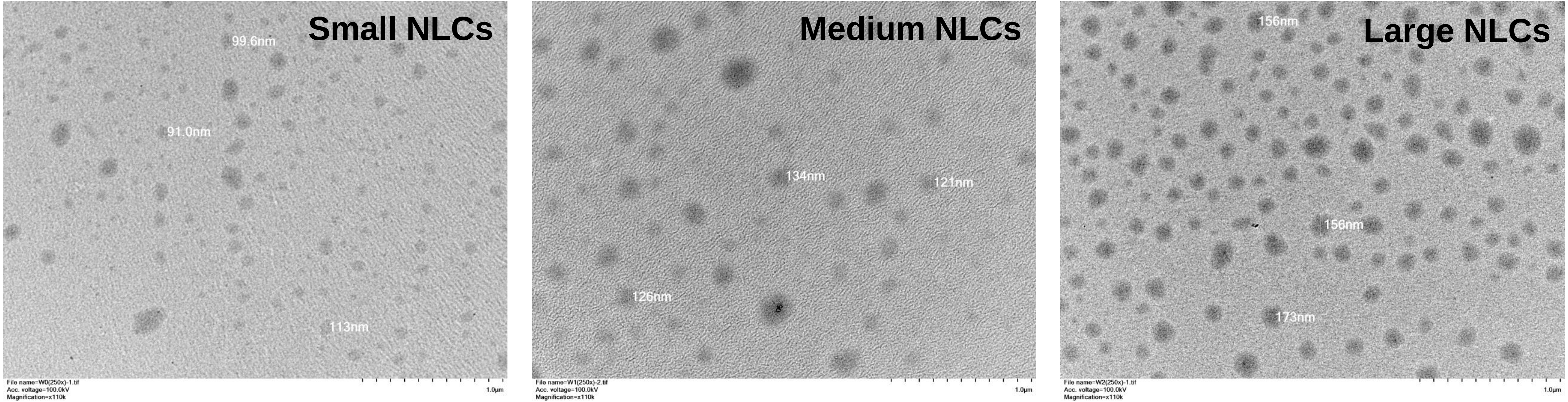
Small NLCs
99.6nm
91.0nm
113nm
File name=W0(250x)-1.tif
Acc. voltage=100.0kV
Magnification=x110k
1.0μm
Medium NLCs
134nm
121nm
126nm
File name=W1(250x)-2.tif
Acc. voltage=100.0kV
Magnification=x110k
1.0μm
Large NLCs
156nm
156nm
173nm
File name=W2(250x)-1.tif
Acc. voltage=100.0kV
Magnification=x110k
1.0μm

Suppl. Fig. 1

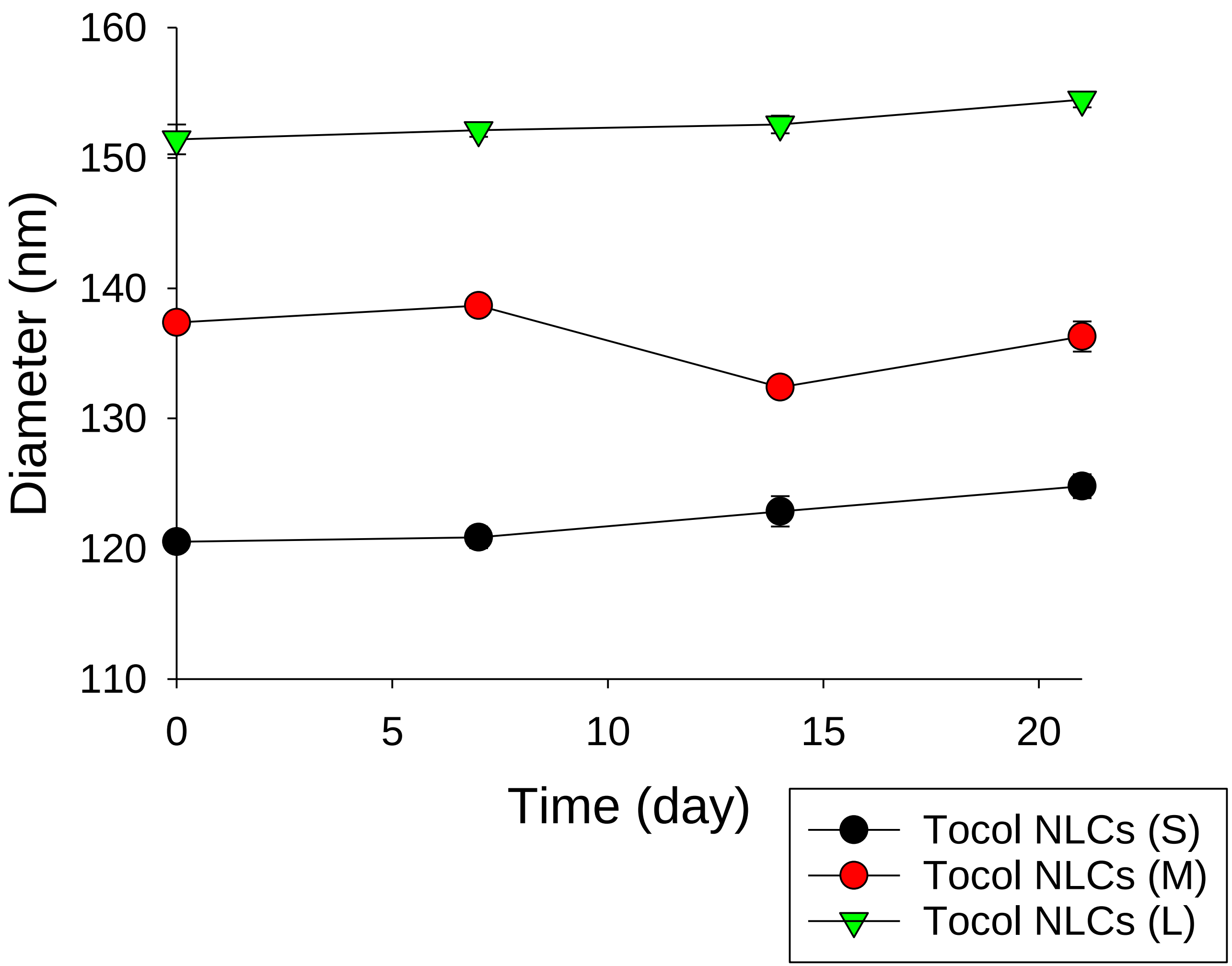

160
150
140
130
120
110
Diameter (nm)
0
5
10
15
20
Time (day)
Tocol NLCs (S)
Tocol NLCs (M)
Tocol NLCs (L)

Suppl. Fig. 2

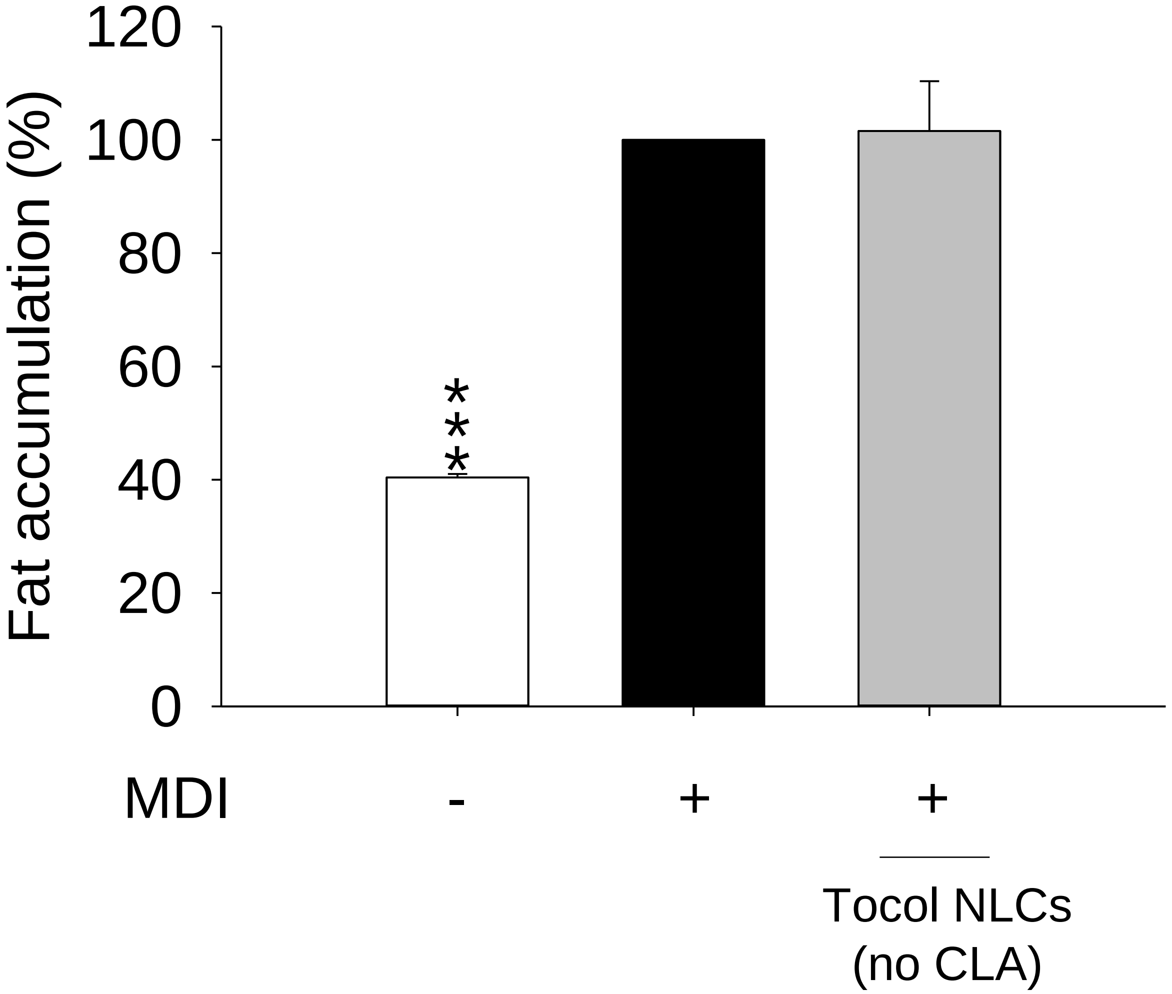

Suppl. Fig. 3

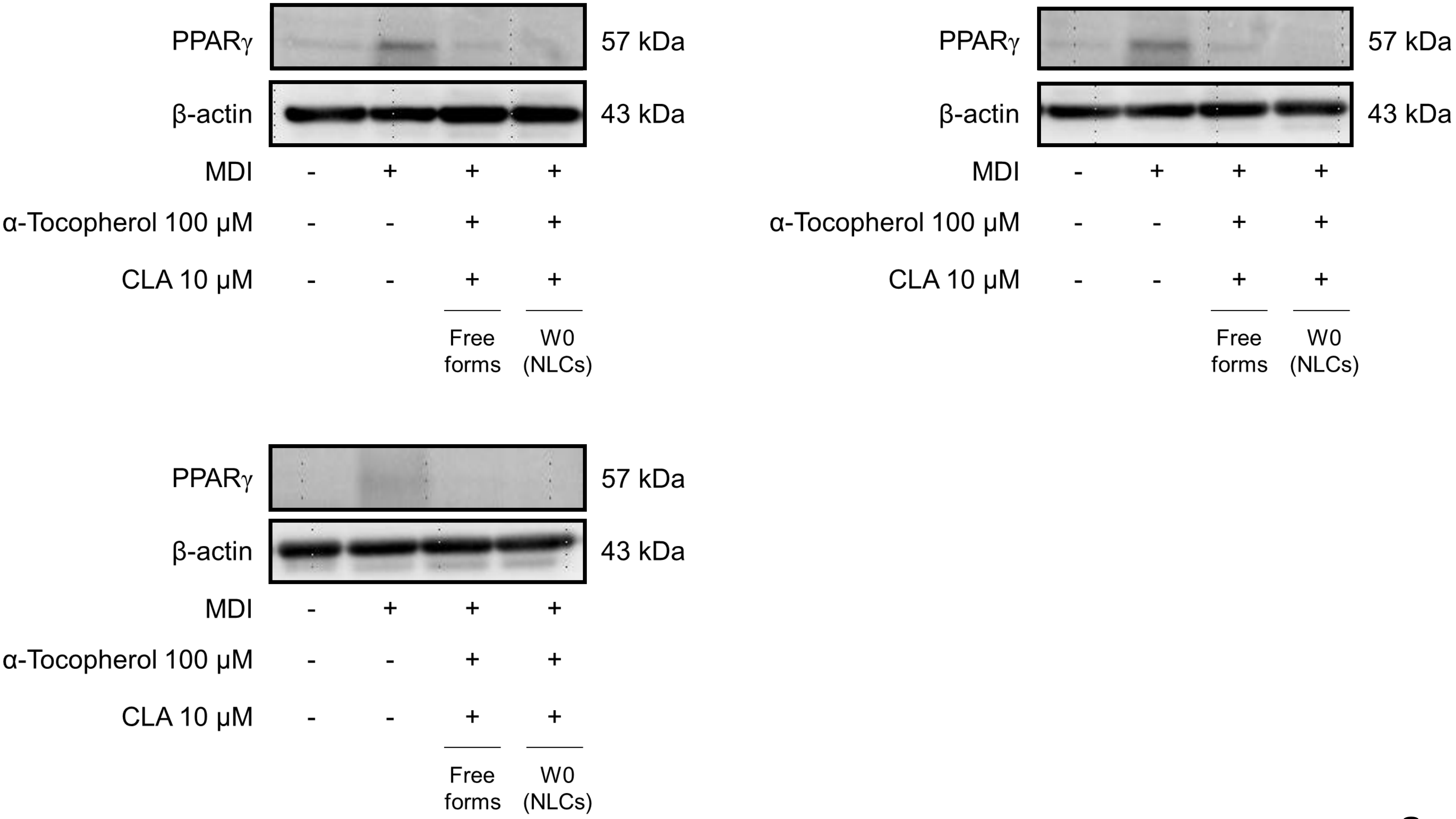
PPARγ
57 kDa
β-actin
43 kDa
MDI - + + +
α-Tocopherol 100 μM - - + +
CLA 10 μM - - + +
Free forms
W0 (NLCs)
PPARγ
57 kDa
β-actin
43 kDa
MDI - + + +
α-Tocopherol 100 μM - - + +
CLA 10 μM - - + +
Free forms
W0 (NLCs)
PPARγ
57 kDa
β-actin
43 kDa
MDI - + + +
α-Tocopherol 100 μM - - + +
CLA 10 μM - - + +
Free forms
W0 (NLCs)

Suppl. Fig. 4A

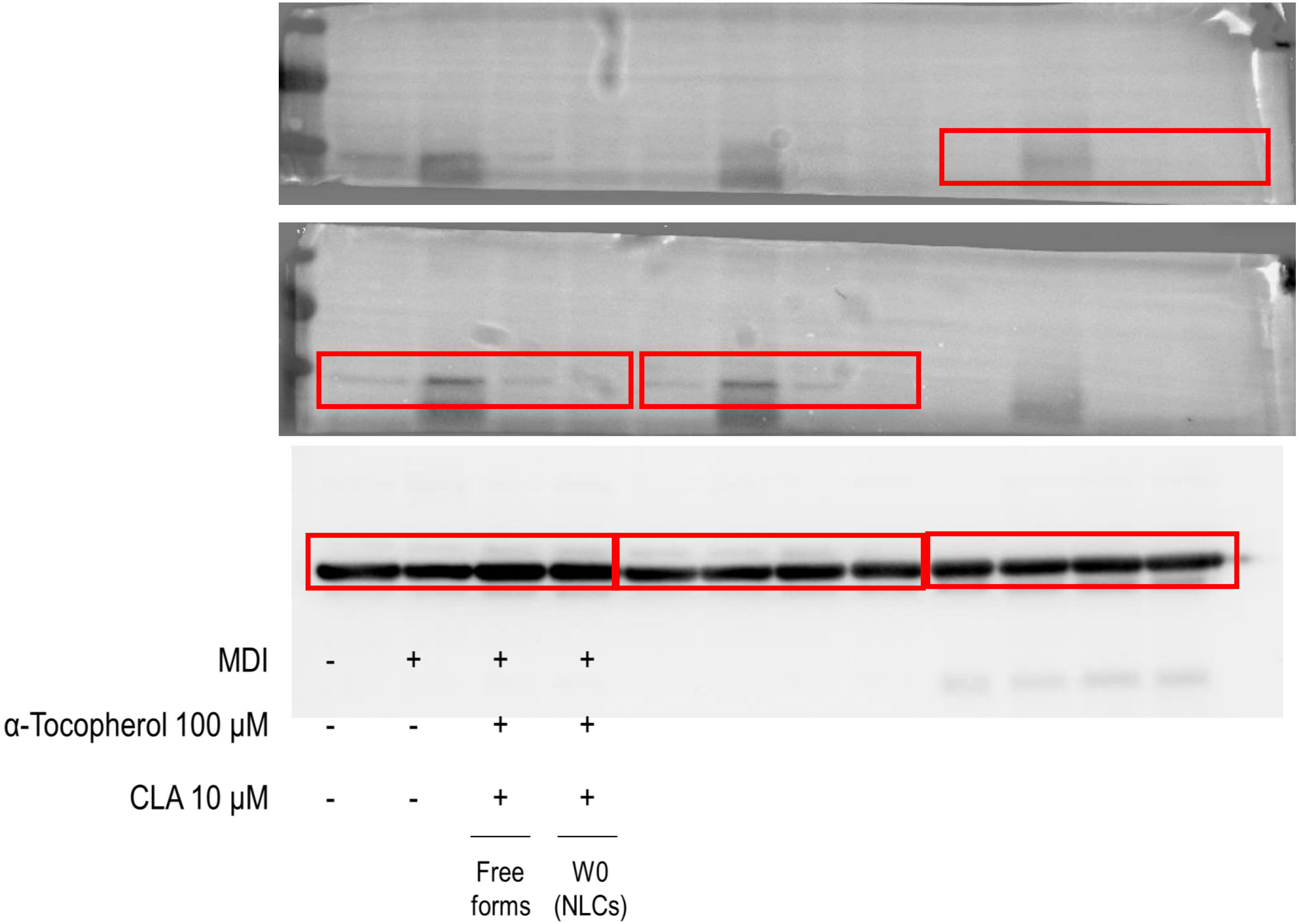

MDI - + + +
α-Tocopherol 100 μM - - + +
CLA 10 μM - - + +
Free forms
W0 (NLCs)

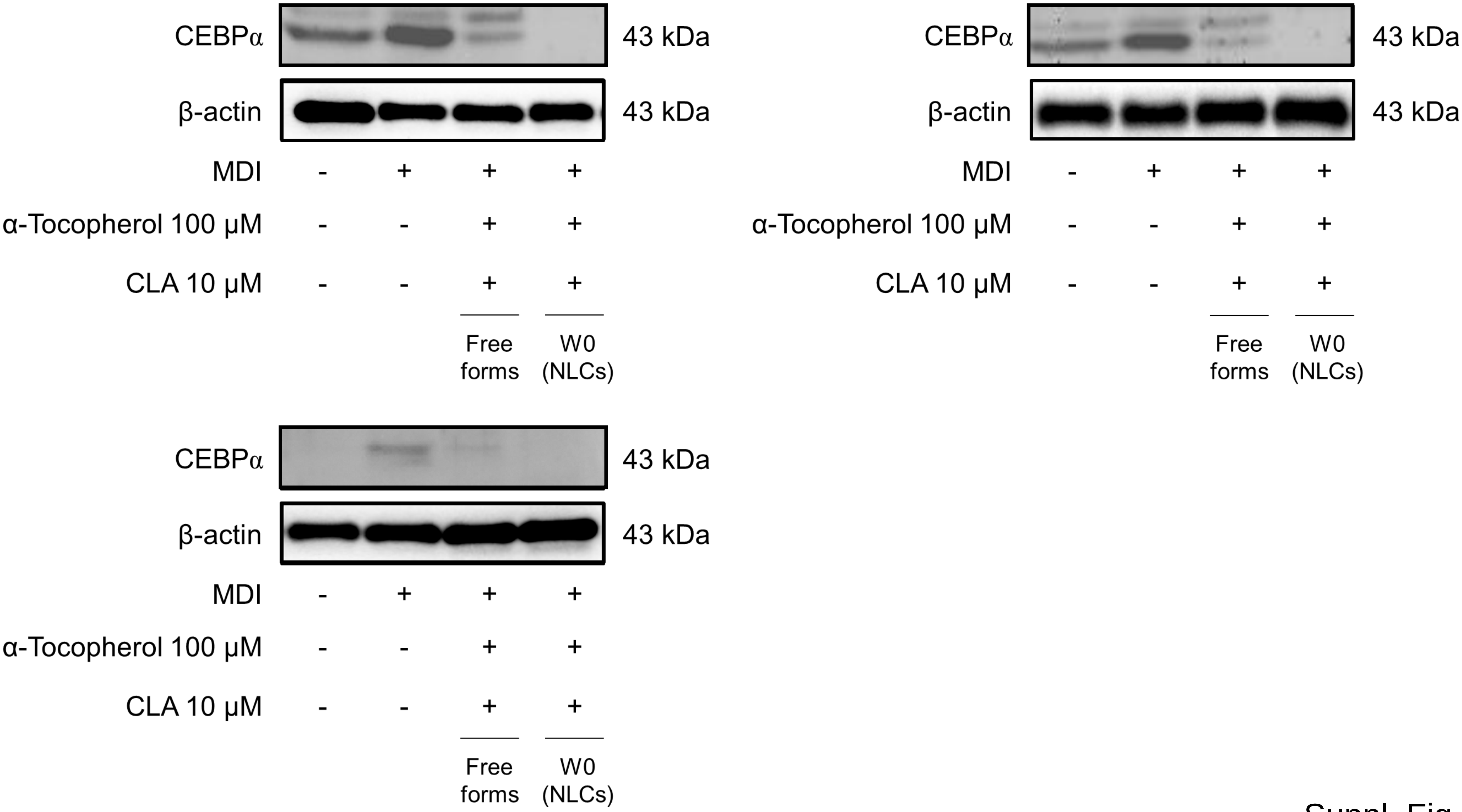
CEBPα
43 kDa
β-actin
43 kDa
MDI - + + +
α-Tocopherol 100 μM - - + +
CLA 10 μM - - + +
Free forms
W0 (NLCs)
CEBPα
43 kDa
β-actin
43 kDa
MDI - + + +
α-Tocopherol 100 μM - - + +
CLA 10 μM - - + +
Free forms
W0 (NLCs)
CEBPα
43 kDa
β-actin
43 kDa
MDI - + + +
α-Tocopherol 100 μM - - + +
CLA 10 μM - - + +
Free forms
W0 (NLCs)

Suppl. Fig. 4B

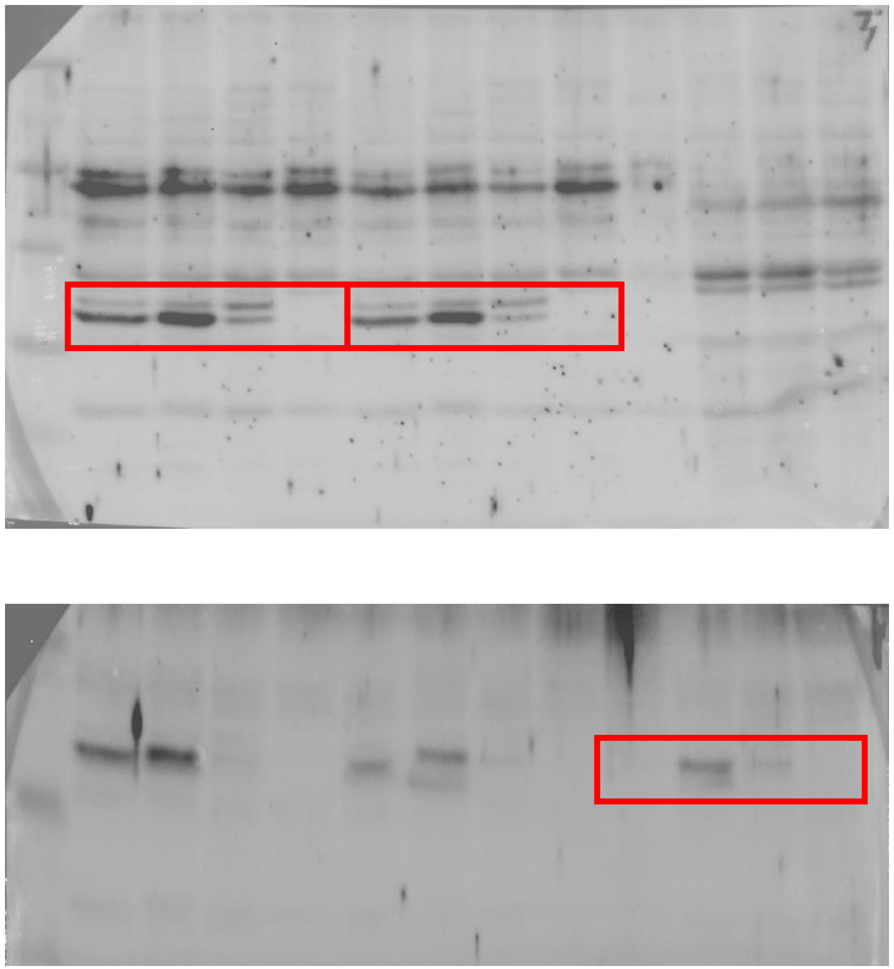

MDI	-	+	+	+
α-Tocopherol 100 μM	-	-	+	+
CLA 10 μM	-	-	+	+
			Free forms	W0 (NLCs)

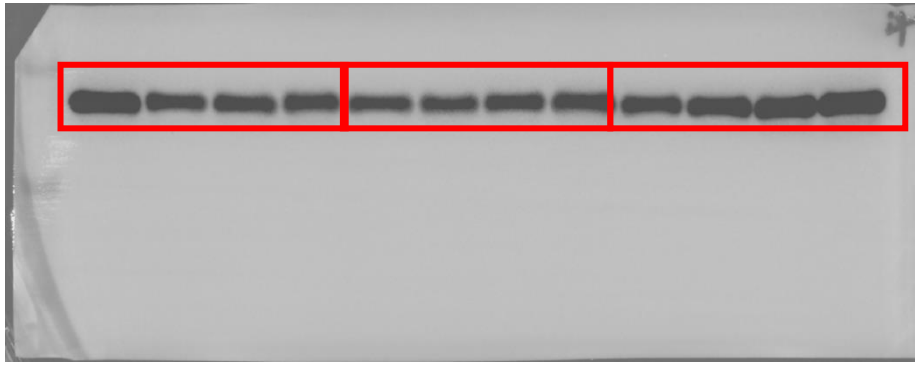

MDI	-	+	+	+
α-Tocopherol 100 μM	-	-	+	+
CLA 10 μM	-	-	+	+
			Free forms	W0 (NLCs)

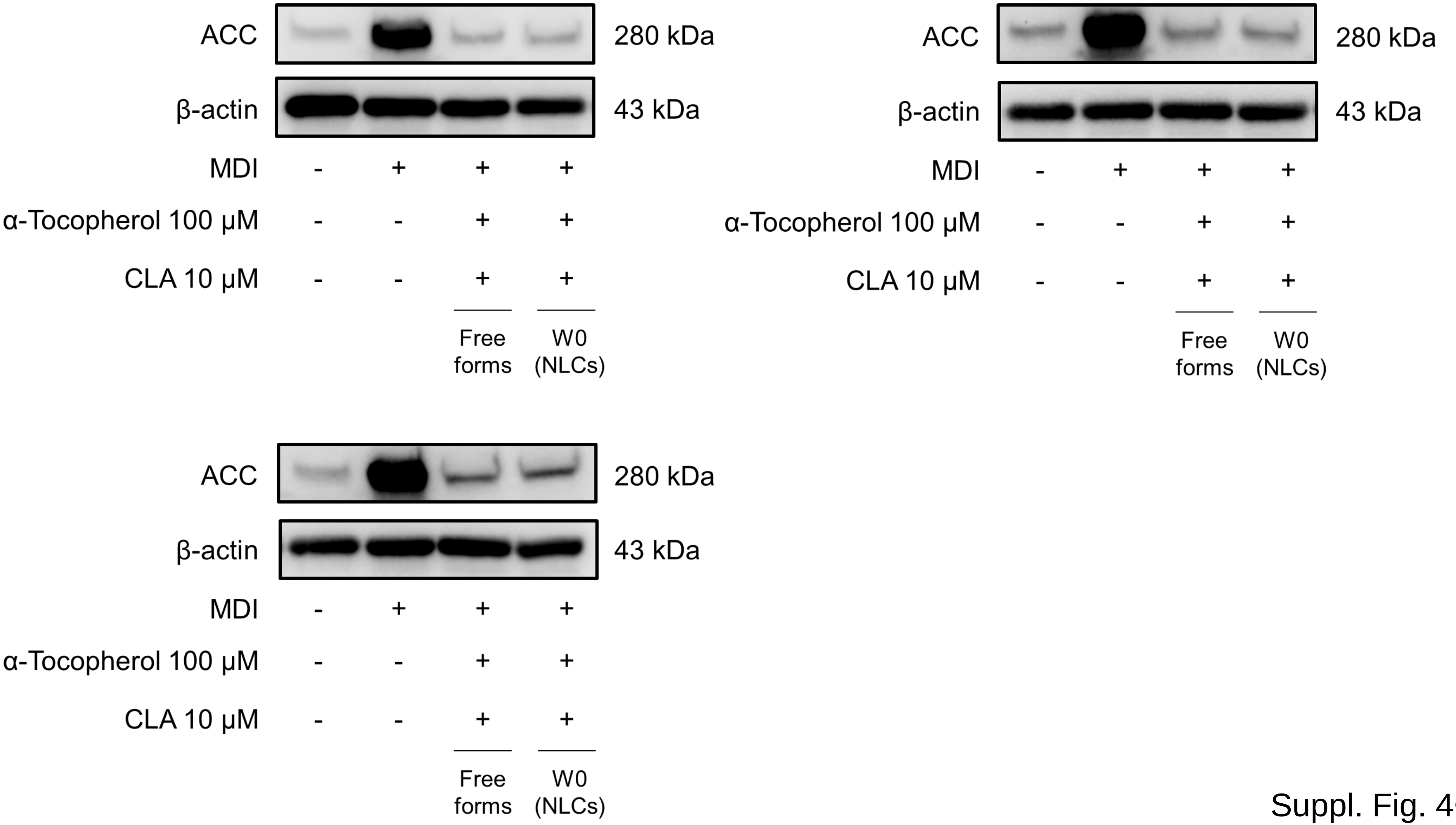
ACC
280 kDa
β-actin
43 kDa
MDI - + + +
α-Tocopherol 100 μM - - + +
CLA 10 μM - - + +
Free forms
W0 (NLCs)
ACC
280 kDa
β-actin
43 kDa
MDI - + + +
α-Tocopherol 100 μM - - + +
CLA 10 μM - - + +
Free forms
W0 (NLCs)
ACC
280 kDa
β-actin
43 kDa
MDI - + + +
α-Tocopherol 100 μM - - + +
CLA 10 μM - - + +
Free forms
W0 (NLCs)

Suppl. Fig. 4C

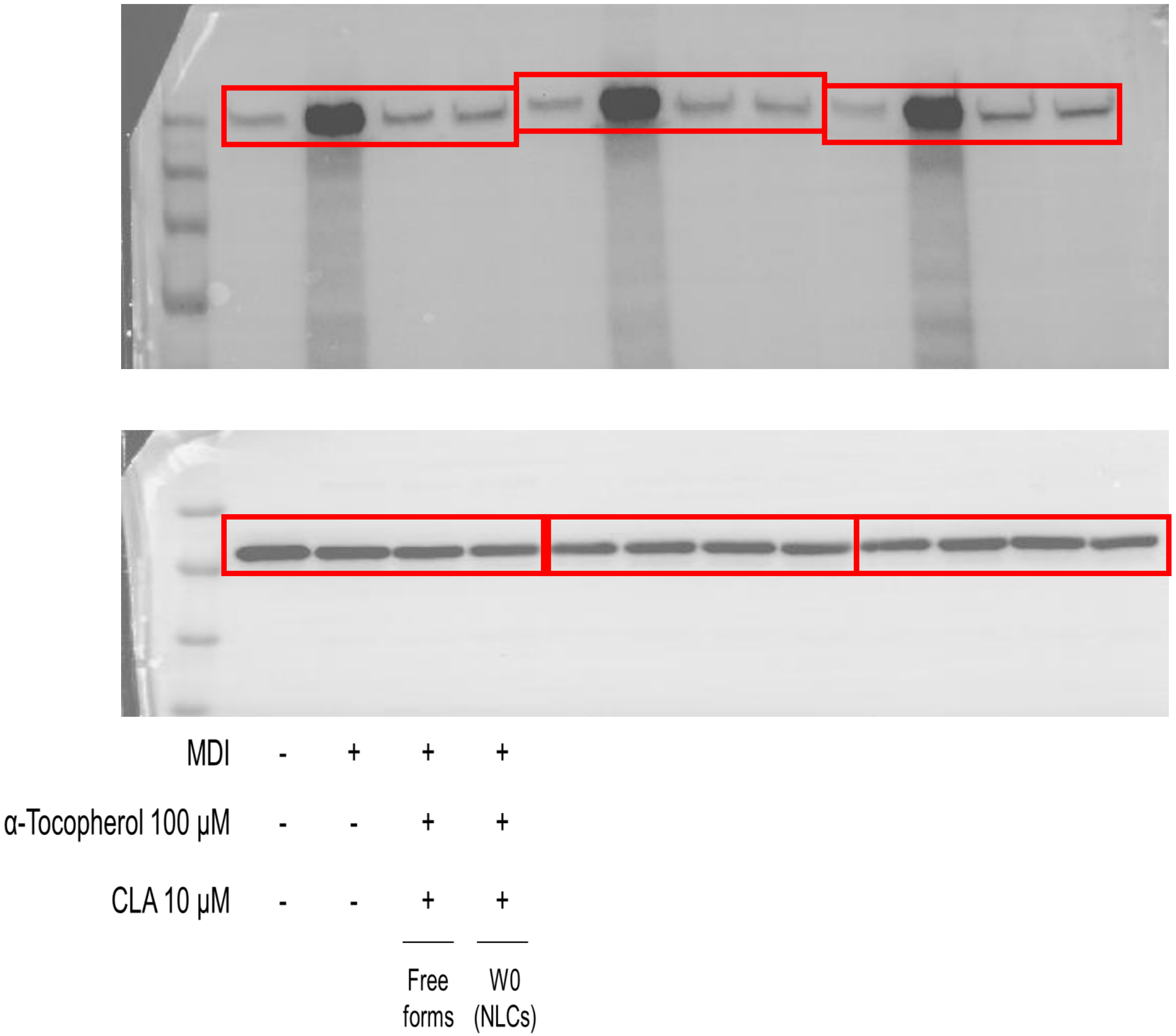
MDI - + + +
α-Tocopherol 100 μM - - + +
CLA 10 μM - - + +
Free forms
W0 (NLCs)

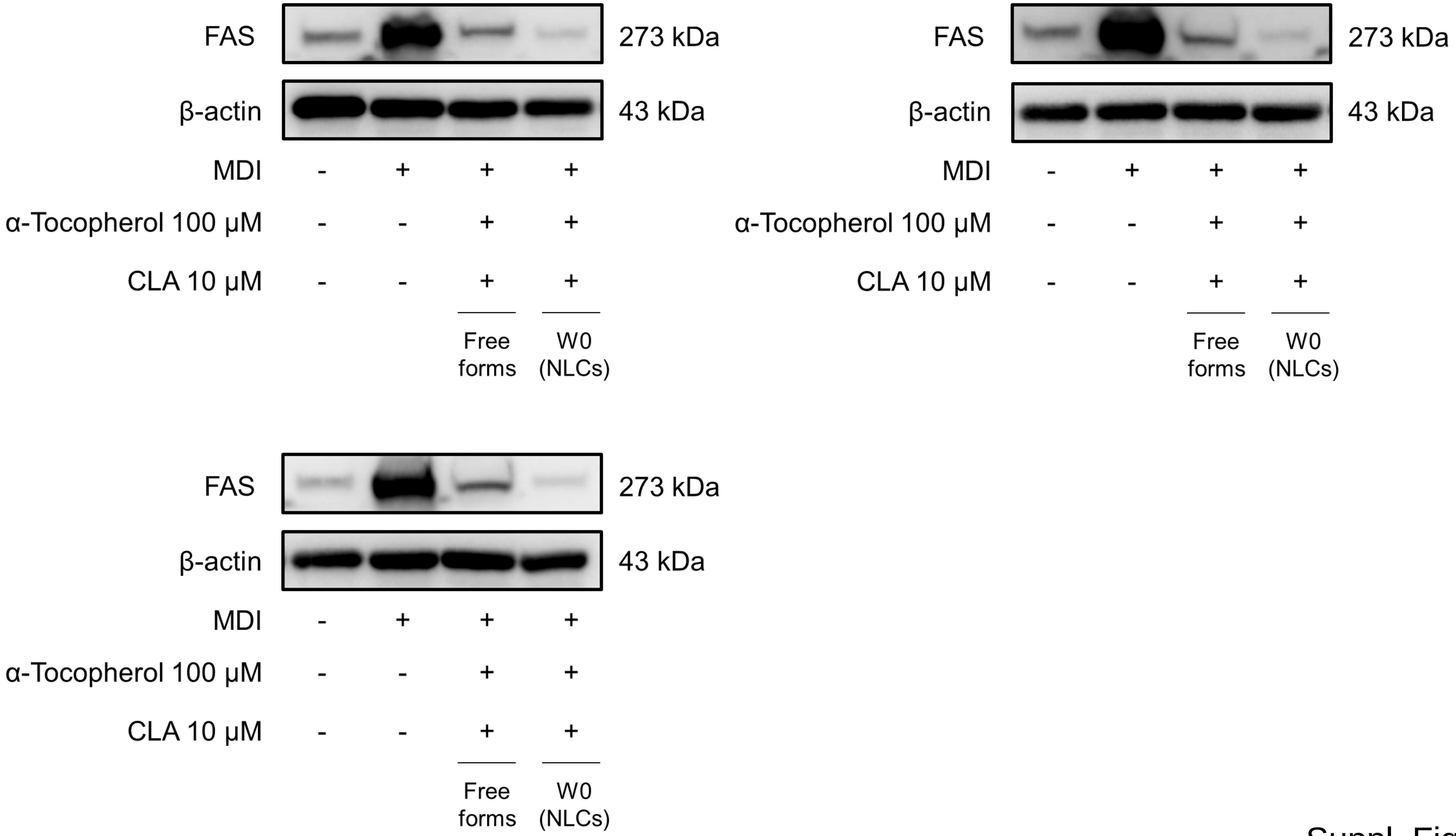
FAS
273 kDa
β-actin
43 kDa
MDI - + + +
α-Tocopherol 100 μM - - + +
CLA 10 μM - - + +
Free forms
W0 (NLCs)
FAS
273 kDa
β-actin
43 kDa
MDI - + + +
α-Tocopherol 100 μM - - + +
CLA 10 μM - - + +
Free forms
W0 (NLCs)
FAS
273 kDa
β-actin
43 kDa
MDI - + + +
α-Tocopherol 100 μM - - + +
CLA 10 μM - - + +
Free forms
W0 (NLCs)

Suppl. Fig. 4D

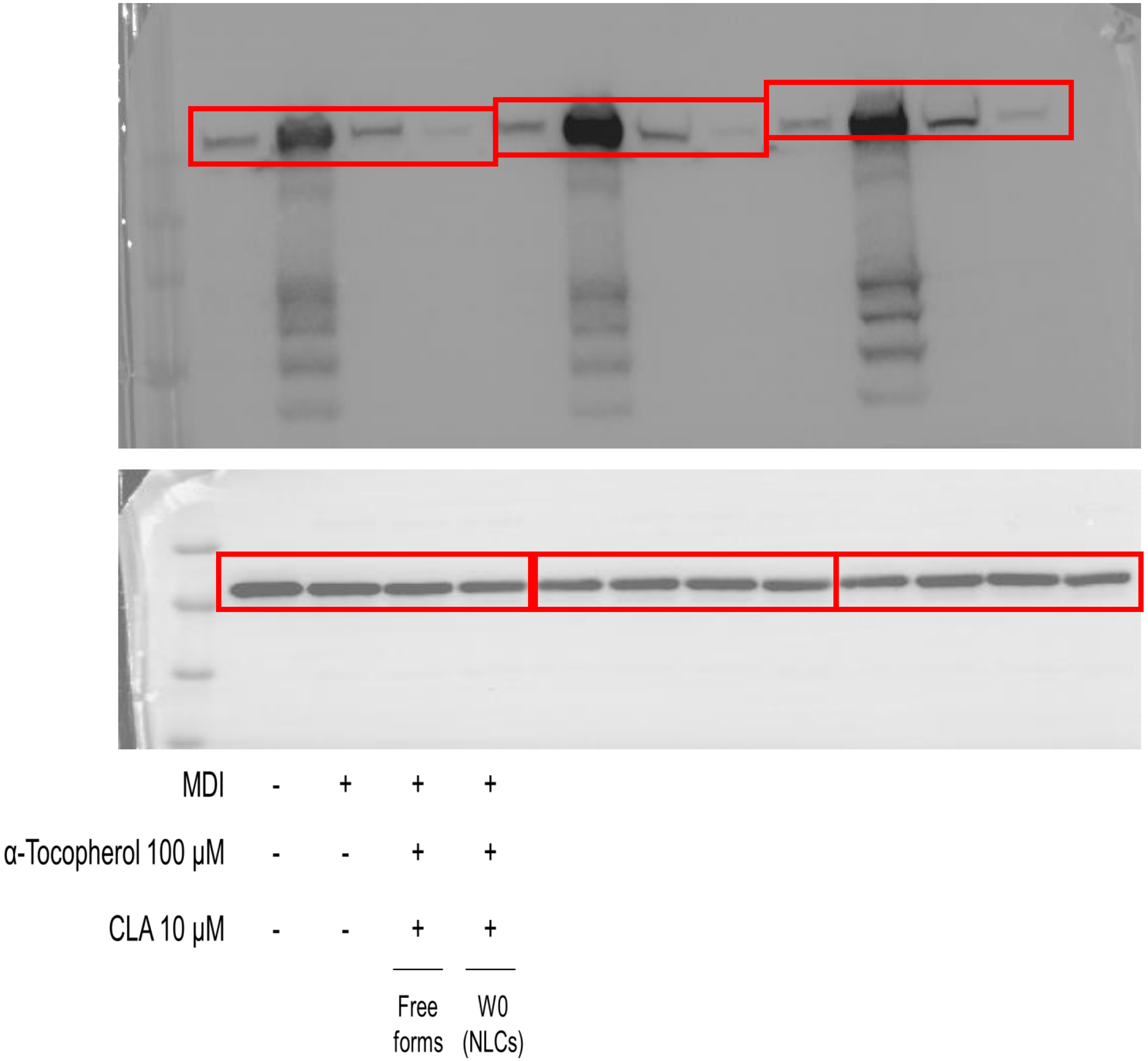
MDI - + + +
α-Tocopherol 100 μM - - + +
CLA 10 μM - - + +
Free forms
W0 (NLCs)

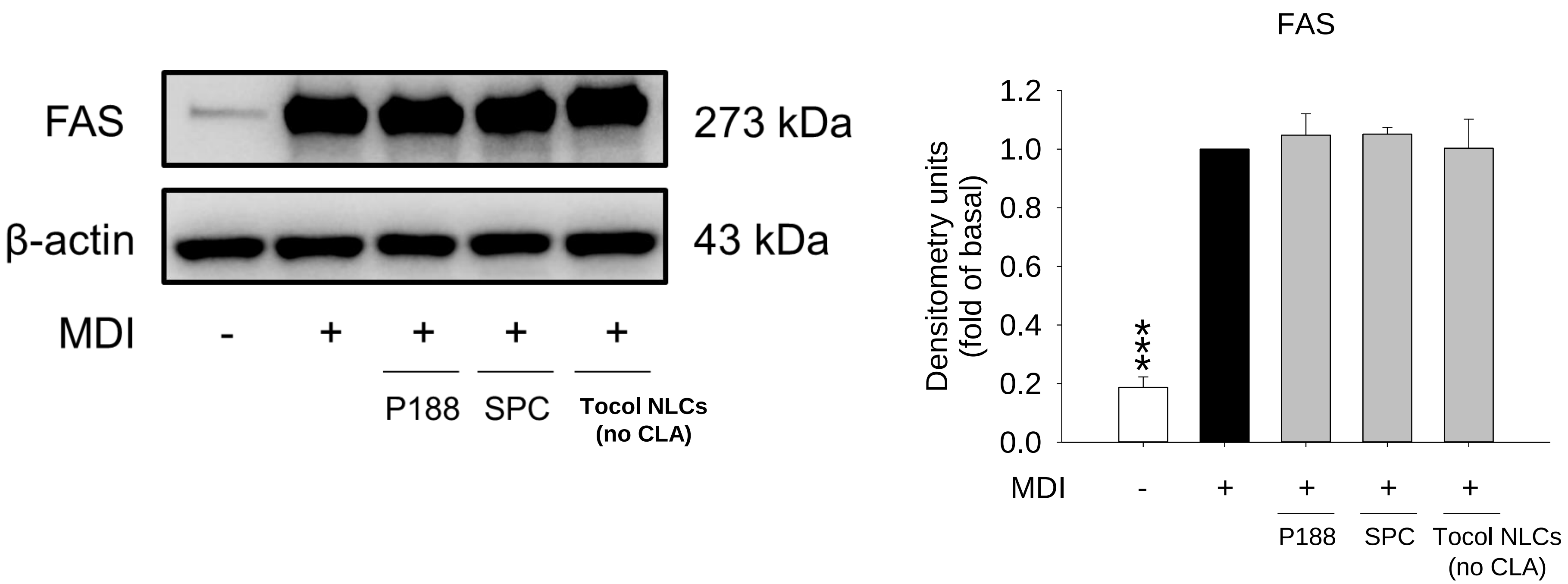
FAS
273 kDa
β-actin
43 kDa
MDI
-
+
+
+
+
P188
SPC
Tocol NLCs
(no CLA)
FAS
Densitometry units
(fold of basal)
1.2
1.0
0.8
0.6
0.4
0.2
0.0
***
MDI
-
+
+
+
+
P188
SPC
Tocol NLCs
(no CLA)

Suppl. Fig. 5
